# Exposure to an acute hypoxic stimulus during early life affects the expression of glucose metabolism-related genes at first-feeding in trout

**DOI:** 10.1038/s41598-017-00458-4

**Published:** 2017-03-23

**Authors:** Jingwei Liu, Elisabeth Plagnes-Juan, Inge Geurden, Stéphane Panserat, Lucie Marandel

**Affiliations:** INRA, Univ Pau & Pays de l’Adour, UMR1419 Nutrition Metabolism and Aquaculture, F-64310, Saint Pée sur Nivelle France

## Abstract

Rainbow trout (*Oncorhynchus mykiss*) is considered a “glucose-intolerant” species. With the aim of programming trout to improve their metabolic use of dietary carbohydrates, we hypothesised that a hypoxic stimulus applied during embryogenesis could later affect glucose metabolism at the first-feeding stage. An acute hypoxic stimulus (2.5 or 5.0 mg·L^−1^ O_2_) was applied for 24 h to non-hatched embryos or early hatched alevins followed by a challenge test with a high carbohydrate diet at first-feeding. The effectiveness of the early hypoxic stimulus was confirmed by the induction of oxygen-sensitive markers such as *egln3*. At first-feeding, trout previously subjected to the 2.5 mg·L^−1^ O_2_ hypoxia displayed a strong induction of glycolytic and glucose transport genes, whereas these glucose metabolism-related genes were affected much less in trout subjected to the less severe (5.0 mg·L^−1^ O_2_) hypoxia. Our results demonstrate that an acute hypoxic stimulus during early development can affect glucose metabolism in trout at first-feeding.

## Introduction

Early life is a critical period of developmental plasticity. During this period, modulations of environmental parameters (e.g., nutrition and oxygen) can affect the phenotype of living organisms, physiologically modifying them in the medium- or long-term of their lifetime and even on a multigenerational scale^1, 2^. This concept is termed “programming”, more specifically, “metabolic programming” when examining the modulation of the metabolism induced by a stimulus received early in life. These “programming effects” on metabolism can be reflected as modified gene expression in critical metabolic pathways in later life^3–6^. Indeed, the long-lasting modification in gene expression pattern is one of the most important biological mechanisms for the persistence of programming effects^7^. The positive aspect of moderate environmental impact (named a stimulus) on phenotypes is now considered to condition livestock to better cope with their future environment, bringing into play their adaptive plasticity. This research aspect has been emerging over a decade in aquaculture and particularly in rainbow trout (*Oncorhynchus mykiss*). The poor use of dietary carbohydrates in this carnivorous species has limited the development of economical plant-based diet formulas, and is a disadvantage for the sustainable development of the salmon farming industry^8–10^.

In this context, programming seems an interesting approach and was explored to try to modify glucose metabolism in trout for the better utilisation of dietary carbohydrates^11, 12^. Recent studies demonstrated for instance that a 5-day high-carbohydrate dietary stimulus at first-feeding resulted in a long-term modification of glucose metabolism-related gene expressions in the muscle and of the intestinal microbiota profile in trout juveniles^12^. However, no persistent programming effects were noted in the liver in juvenile fish, despite the liver’s pivotal role in glucose metabolism, which could be related to the experimental conditions used for programming. In addition to the importance of the type of the stimulus applied, another decisive point in the concept of programming is that the susceptibility of organisms may be limited to a critical period (a window of metabolic plasticity) during which an environmental stimulus may impact durably on metabolism^13^. In trout juveniles two of the five genes encoding the glucose-6-phosphatase (G6pc), the enzyme catalysing the last step of gluconeogenesis, were shown to be atypically up-regulated by dietary carbohydrates^14, 15^, and this phenotype was demonstrated to take place as soon as the first-feeding stages^15^. These results suggest that applying a stimulus at first-feeding, as Geurden *et al*. did^12^, may be too late to programme glucose metabolism (or at least gluconeogenesis) in trout for the long-term. Moreover, these results established the premise that first-feeding can be considered the earliest period at which the preliminary outcome of metabolic programming can be assessed.

Here we hypothesised that applying a stimulus before first-feeding, *i*.*e*., during embryogenesis, may be more efficient for programming glucose metabolism in rainbow trout. Fish embryos and larvae depend exclusively on their endogenous yolk reserves until the first-feeding. Therefore, a non-nutritional stimulus (*i*.*e*., temperature or hypoxia) appears to be an ideal choice, because embryos of the oviparous trout are directly exposed to environmental variations. In the present study, hypoxia was chosen as a stimulus for several reasons. Oxygen is closely linked to glucose metabolism in aerobic organisms^16^. Hypoxia can trigger a shift in metabolism from mitochondrial oxidative phosphorylation to an anaerobic glycolytic pathway in mammals^17^. Similarly, hypoxia is known to modulate glucose metabolism in several fish species, including rainbow trout^18–26^. Although no data relating to programming assays with hypoxia in trout are available, previous studies of mammals have found that intra-uterine hypoxia could inhibit gluconeogenesis and alter plasma glucose content in offspring^6, 27^, demonstrating that hypoxia has a long-lasting programming effect on glucose metabolism. Moreover, such a stimulus is easy to apply on a large scale to individuals compared to microinjections performed directly in the yolk as previously tested in zebrafish^28^. We thus hypothesise that an early exposure to hypoxia in trout may modulate glucose metabolism and that such a modification could be recorded as a “physiological memory” and reveal itself later in life when fish are challenged with a high carbohydrate diet. The stimulus duration was set as a 24 h acute hypoxic stimulus as trout are considered as an oxygen-sensitive species^29^, that are susceptible to chronic hypoxia with possible adverse consequences on growth performance^30, 31^.

Another important factor in a nutritional programming strategy is the time point at which the stimulus is applied. A previous study in trout conducted during ontogenesis revealed that mRNA levels of genes involved in glucose metabolism increased in embryos during the development of the primitive liver (approximately 160 °D, degree day)^15^. Hence, a stimulus applied just before this stage could be a promising time point to programme glucose metabolism. However, before hatching, the presence of the chorion protects the embryo from external environmental changes^32^ and may function as a barrier for oxygen diffusion^31, 33^. It is therefore uncertain whether an acute 24 h hypoxic stimulus applied at this stage can affect the oxygen availability inside the chorion. Hence, an additional stage was selected for applying the stimulus: between hatching and first-feeding (552 °D).

In summary, the present study proposes to investigate the short-term effects of an early acute hypoxic stimulus applied at two different developmental stages on glucose metabolism in trout by challenging with a high carbohydrate diet at the first-feeding. As transcriptional modification is the first step to mediate metabolic programming, we mainly focus on the programming effects at the transcriptional level, *i*.*e*., alteration in mRNA levels of critical genes involved in glycolysis, gluconeogenesis and the glucose transport pathway.

## Results

### Survival of embryos and alevins

In the present study, the hypoxic stimulus applied at the embryo stage (152 °D) did not affect the survival and malformation monitored later in life (2 days after the hypoxic stimulus, at hatching and at first-feeding, Table 1). However, there was a decrease in the survival rates in fish subjected to the hypoxic stimulus at the alevin stage (552 °D) both after the hypoxic stimulus and before first-feeding. The mortality increased significantly with the elevation of the hypoxia level (*p* < 0.05). In addition, fish which were subjected to hypoxia at 552 °D displayed a significant decrease in the malformation (*p* < 0.05).

**Table 1:** Survival of embryos and alevins after hypoxic stimulus, at hatching and before first-feeding.

Stimulus stage	Hypoxia level	2 days after hypoxic stimulus	At hatching	Before first-feeding	
		Survival	Survival	Survival	Malformation
Embryos (152 °D)	2.5 mg·L^−1^ O_2_	99.1 ± 0.4%	96.3 ± 0.8%	76.3 ± 2.0%	5.6 ± 2.2%
	5.0 mg·L^−1^ O_2_	95.4 ± 0.5%	95.5 ± 1.1%	76.7 ± 2.7%	5.6 ± 2.0%
	Normoxia	99.2 ± 0.4%	94.9 ± 0.7%	75.0 ± 2.8%	4.5 ± 1.6%
Alevins (552 °D)	2.5 mg·L^−1^ O_2_	41.9 ± 7.2%^a^		41.4 ± 6.8%^a^	1.3 ± 1.3%^a^
	5.0 mg·L^−1^ O_2_	65.1 ± 7.2%^b^		64.6 ± 7.0%^b^	0.9 ± 0.6%^a^
	Normoxia	78.5 ± 3.8%^c^		75.0 ± 2.8%^c^	4.5 ± 1.6%^b^

Data are represented by means (n = 6), data bearing the same superscripts within a column were not significantly different (*p* > 0.05, Pearson’s Chi-squared test).

### *In silico* analysis of hypoxia-sensitive and glucose metabolism-related genes

By analysing the rainbow trout genome^34^, we identified for the first time several genes sharing high sequence homology with the hypoxia-sensitive and some of the glucose metabolism-related mammalian orthologues. One gene is related to *Pdk1* (pyruvate dehydrogenase kinase 1, which is responsible for the inactivation of the enzyme pyruvate dehydrogenase, leading to the blockade of pyruvate conversion into acetyl-coA). Two genes are related to each of the following mammalian genes: *Hif1α* (Hypoxia inducible factor 1 alpha, the master transcriptional factor involved in hypoxic responses), *Ldha* (Lactate dehydrogenase A, catalyses the conversion of L-lactate and NAD to pyruvate and NADH in the final step of anaerobic glycolysis), *Slc16a3* (solute carrier family 16 (monocarboxylic acid transporters), member 3, which meditates lactic acid transport across plasma membranes), *Slc2a2* (solute carrier family 2 (facilitated glucose transporter), member 2, also called *Glut2*), *Slc2a4* (solute carrier family 2 (facilitated glucose transporter), member 4, also called *Glut4*) *and Egln3* (egl-9 family hypoxia-inducible factor 3, cellular oxygen sensor, which catalyses the degradation process of Hif1α protein, but is also a downstream gene of Hif1). Finally, four genes each are related to *Slc2a1* (solute carrier family 2 (facilitated glucose transporter), member 1, also called *Glut1*), *Pfkm* (phosphofructokinase, muscle, which catalyses the phosphorylation of fructose-6-phosphate to fructose-1,6-bisphosphate in glycolysis) and *Pkm* (pyruvate kinase, muscle, which catalyses the transformation of phosphoenolpyruvate to pyruvate in glycolysis) genes. A syntenic analysis and when necessary a phylogenetic analysis, using full-length vertebrate protein sequences were then performed to clarify the identity of these rainbow trout sequences. These results are presented in the Supplementary File; final annotations of the different genes can be found in Supplementary Table S1. The *in silico* analyses were performed in previous studies^14, 15^ for *Gck* (glucokinase, responsible for the phosphorylation glucose to glucose-6-phosphate), *Pfkl* (phosphofructokinase, liver, which catalyses the phosphorylation of fructose-6-phosphate to fructose-1,6-bisphosphate in glycolysis), *Pkl* (pyruvate kinase, liver, which catalyses the transform of phosphoenolpyruvate to pyruvate in glycolysis), *Pck* (phosphoenolpyruvate carboxykinase, cytosolic *Pck*1 and mitochondrial *Pck*2, which catalyses the conversion of oxaloacetate to phosphoenolpyruvate), *Fbp* (fructose-1,6-bisphosphatase, which catalyses the hydrolysis of fructose 1,6-bisphosphate to fructose 6-phosphate), and *G6pc* (glucose-6-phosphatase, which catalyses the hydrolysis of D-glucose 6-phosphate to D-glucose).

### Expression analysis of targeted hypoxia-sensitive genes at the end of the acute hypoxic stimulus in both embryos and alevins

**Figure 1:**
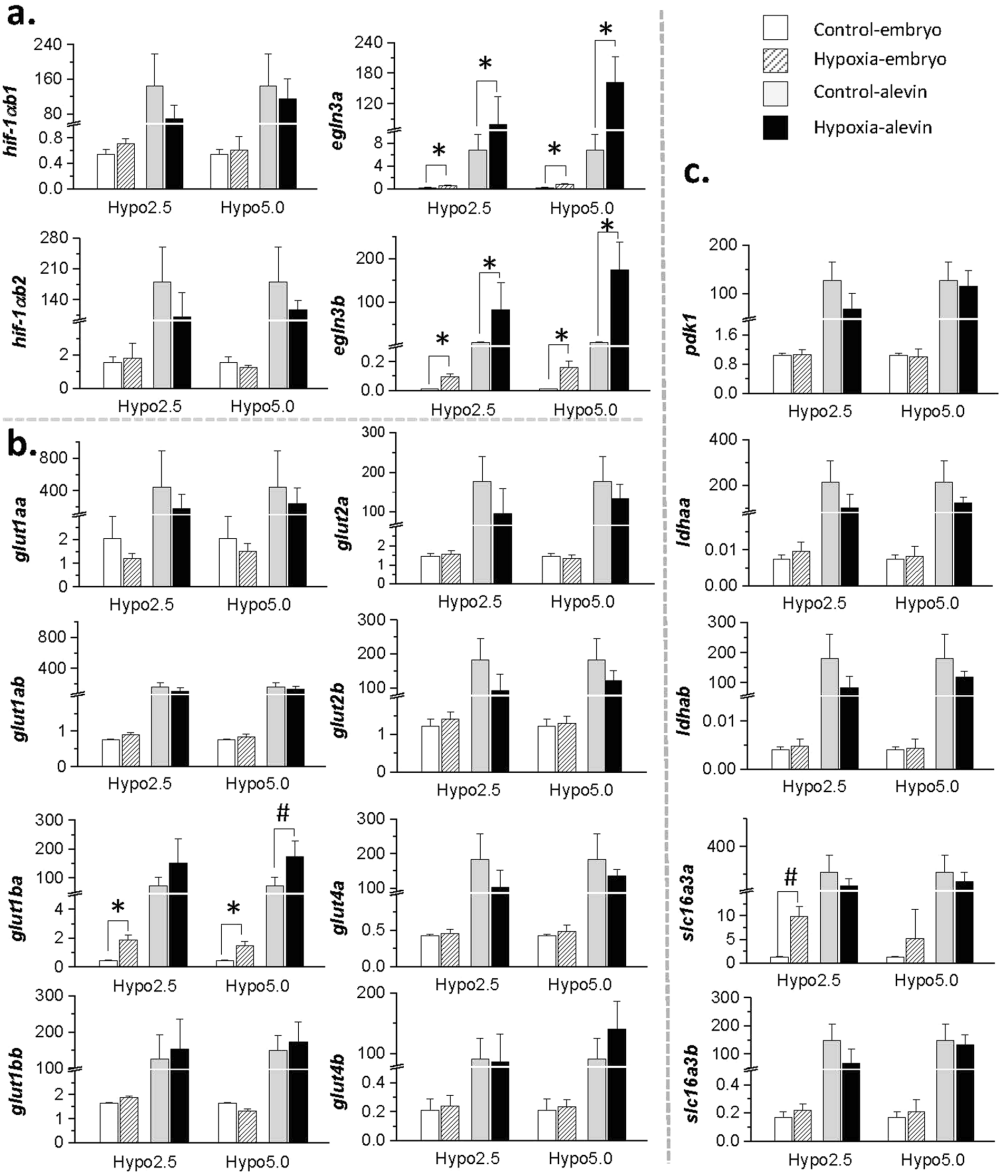
Molecular confirmation of the effect of the hypoxic stimulus on targeted oxygen-sensitive genes in non-hatched embryos and early hatched alevins. The relative abundance of mRNA of (**a**) hypoxia-responsive genes, (**b**) glucose transport genes and (**c**) pyruvate conversion and lactate transport genes in embryos (kept under normoxia, white bars; exposed to hypoxia, slash bars) and in alevins (kept under normoxia, grey bars; exposed to hypoxia, black bars). Data are presented as the mean ± SD (n = 6), significant differences were evaluated by nonparametric Kruskal-Wallis test followed by a Tukey’s test (^#^ *p* < 0.05; *p < 0.001).

To ensure the effectiveness of hypoxia, qPCR was performed to determine the mRNA levels of several hypoxia-related genes in the whole body of embryos and alevins at the end of the 24 h hypoxic stimulus. Compared with the normoxia group, trout that were exposed to the 24 h hypoxic stimulus displayed significantly higher mRNA levels of *egln3* ohnologous genes (*p* < 0.001), in both stages at which the stimulus was applied and both levels of hypoxia (Fig. 1a). Similar results were found for one *glut1* gene, *glut1ba*, which displayed an increase in its mRNA level in trout subjected to hypoxia at the embryo stage at both hypoxic levels (2.5 mg·L^−1^ O_2_ or 5.0 mg·L^−1^ O_2_, *p* < 0.001). Additionally, trout exposed to a 5.0 mg·L^−1^ O_2_ hypoxic stimulus at the alevin stage displayed a higher mRNA level of *glut1ba* compared with control fish (Fig. 1b, *p* < 0.05). In addition, at the embryo stage, the mRNA level of *slc16a3a* was significantly higher in trout exposed to the 2.5 mg·L^−1^ O_2_ hypoxic stimulus compared with those in the normoxic condition (Fig. 1c, *p* < 0.05). No significantly differences were found in the mRNA levels of *hif1α*, *glut2* and *glut4* ohnologues, *pdk1*, *ldha* duplicated genes and *slc16a3b* gene among treatments.

### Expression analysis of targeted hypoxia-sensitive egln3 ohnologs at first-feeding

The mRNA levels of *egln3a* and *egln3b* were measured in trout at first-feeding (Table 2). There was no significant difference in *egln3b* mRNA levels in trout regardless of the treatment. In trout subjected to 5.0 mg·L^−1^ O_2_ hypoxia, the *egln3a* mRNA level remained stable irrespective of the diet they received. However, in trout exposed to the 2.5 mg·L^−1^ O_2_ hypoxic stimulus at both stages, the *egln3a* mRNA level was significantly higher in trout fed the HC (high carbohydrate) diet than those fed the NC (no carbohydrate) diet at first feeding (*p* < 0.05). Additionally, trout subjected to 2.5 mg·L^−1^ O_2_ hypoxic stimulus at the alevin stage exhibited significantly higher *egln3a* mRNA level than normoxic groups (Table 2, *p* < 0.05).

**Tables 2:** Effects of early hypoxia and high-carbohydrate diet at first-feeding on mRNA level of *egln3a* and *egln3b*.

Target gene	Hypoxia stage	Hypoxia level: 2.5 mg·L^−1^							Target gene	Hypoxia stage	Hypoxia level: 5.0 mg·L^−1^						
		N-NC	N-HC	H-NC	H-HC	*p* of Two-way *ANOVA*					N-NC	N-HC	H-NC	H-HC	*p* of Two-way *ANOVA*		
		Means ± SD	Means ± SD	Means ± SD	Means ± SD	Diet	Oxygen	D*O			Means ± SD	Means ± SD	Means ± SD	Means ± SD	Diet	Oxygen	D*O
*egln3a*	Embryo	1.96 ± 1.66	3.89 ± 4.17	2.89 ± 1.67	6.90 ± 1.19	**0**.**01**	0.07	0.32	*egln3a*	Embryo	2.87 ± 2.07	4.20 ± 4.10	1.80 ± 1.97	3.30 ± 3.08	0.25	0.42	0.94
	Alevin			3.82 ± 1.42	8.05 ± 3.07	**0**.**01**	**0**.**02**	0.33		Alevin			2.67 ± 2.53	4.74 ± 4.87	0.26	0.91	0.80
*egln3b*	Embryo	2.79 ± 2.90	3.51 ± 3.57	3.64 ± 1.72	5.02 ± 1.22	0.32	0.27	0.75	*egln3b*	Embryo	4.07 ± 3.44	3.74 ± 3.44	2.76 ± 2.29	3.19 ± 2.46	0.97	0.45	0.76
	Alevin			3.84 ± 0.60	5.52 ± 1.90	0.26	0.15	0.64		Alevin			3.22 ± 2.42	4.18 ± 3.83	0.82	0.88	0.64

N-NC, N-HC, H-NC and H-HC stands for fish subjected to normoxia and no carbohydrate diet, normoxia and high carbohydrate diet, hypoxia and no carbohydrate diet and hypoxia and high carbohydrate diet, respectively. Data were presented as mean ± SD (n = 6), statistical differences were evaluated by two-way *ANOVA* (*p* < 0.05, values in bold).

### Expression analysis of glucose metabolism-related genes at first-feeding

#### Genes involved in glycolysis pathway

At the time of first-feeding, mRNA levels of the glycolytic genes *gcka*, *gckb* and *pfkmba* were increased in trout fed the HC diet (Table 3, *p* < 0.05), regardless of the stage (embryo *vs* alevin) or the level of hypoxic stimulus. Compared with the normoxic group, the mRNA levels of most of the glycolytic genes (*pfkla*, *pfklb*, *pfkmaa*, *pfkmbb*, *pkmaa*, *pkmab*, *pkmba* and *pkmbb*) were significantly increased in trout subjected to a high level of hypoxia (2.5 mg·L^−1^ O_2_) at the embryo stage or the alevin stage (*p* < 0.05). However, the mRNA levels of *gckb* and *pfkmab* were only increased in trout subjected to the 2.5 mg·L^−1^ O_2_ hypoxic stimulus at the alevin stage (*p* < 0.05), but not at the embryo stage. By contrast, the low level of hypoxia (5.0 mg·L^−1^ O_2_) applied during the early stimulus failed to affect the mRNA levels of glycolytic genes. Interestingly, an interaction between the diet received at first-feeding (*i*.*e*., HC or NC diet) and the oxygen level during the early stimulus (*i*.*e*., normoxia *vs* hypoxia) was observed for *gckb* (*p* = 0.04) in trout subjected to the 2.5 mg·L^−1^ O_2_ hypoxic stimulus at the alevin stage (Table 3).

**Table 3:** Effects of early hypoxia and high-carbohydrate diet at first-feeding on gene expressions involved in glycolysis pathway.

Target gene	Hypoxia stage	Hypoxia level: 2.5 mg·L^−1^							Target gene	Hypoxia stage	Hypoxia level: 5.0 mg·L^−1^						
		N-NC	N-HC	H-NC	H-HC	*p* of Two-way *ANOVA*					N-NC	N-HC	H-NC	H-HC	*p* of Two-way *ANOVA*		
		Means ± SD	Means ± SD	Means ± SD	Means ± SD	Diet	Oxygen	D*O			Means ± SD	Means ± SD	Means ± SD	Means ± SD	Diet	Oxygen	D*O
*gcka*	Embryo	0.02 ± 0.01	4.19 ± 2.88	0.03 ± 0.01	4.40 ± 2.50	**0**.**00**	0.88	0.90	*gcka*	Embryo	0.02 ± 0.02	5.97 ± 4.97	0.04 ± 0.03	2.47 ± 1.55	**0**.**00**	0.12	0.11
	Alevin			0.02 ± 0.00	5.60 ± 2.05	**0**.**00**	0.34	0.34		Alevin			0.02 ± 0.02	5.37 ± 4.82	**0**.**00**	0.84	0.83
*gckb*	Embryo	0.03 ± 0.02	5.14 ± 4.01	0.17 ± 0.15	7.77 ± 5.04	**0**.**00**	0.31	0.36	*gckb*	Embryo	0.10 ± 0.08	5.83 ± 4.71	0.10 ± 0.08	3.77 ± 2.72	**0**.**00**	0.37	0.37
	Alevin			0.07 ± 0.01	11.03 ± 5.19	**0**.**00**	**0**.**04**	**0**.**04**		Alevin			0.09 ± 0.07	5.09 ± 4.60	**0**.**00**	0.78	0.79
*pfkla*	Embryo	2.54 ± 1.69	3.33 ± 2.69	5.49 ± 3.03	4.52 ± 1.46	0.92	**0**.**04**	0.36	*pfkla*	Embryo	3.41 ± 2.41	3.69 ± 2.98	2,61 ± 1.71	2.32 ± 1.59	0.99	0.25	0.76
	Alevin			5.11 ± 0.97	5.01 ± 1.31	0.65	**0**.**01**	0.55		Alevin			3.16 ± 2.24	3.16 ± 2.69	0.90	0.72	0.90
*pfklb*	Embryo	2.95 ± 1.84	4.15 ± 3.14	6.22 ± 2.65	5.35 ± 0.66	0.86	**0**.**03**	0.28	*pfklb*	Embryo	3.17 ± 2.18	3.63 ± 2.77	3.16 ± 2.56	2.27 ± 1.60	0.82	0.48	0.48
	Alevin			5.25 ± 0.47	5.43 ± 0.59	0.38	**0**.**03**	0.50		Alevin			3.08 ± 2.12	3.07 ± 2.43	0.82	0.74	0.81
*pfkmaa*	Embryo	1.53 ± 1.14	3.12 ± 2.26	4.01 ± 1.95	4.84 ± 1.11	0.10	**0**.**01**	0.59	*pfkmaa*	Embryo	2.13 ± 1.43	3.39 ± 2.47	1.83 ± 1.25	2.97 ± 2.25	0.14	0.65	0.94
	Alevin			3.91 ± 0.72	4.70 ± 0.97	0.05	**0**.**00**	0.50		Alevin			2.25 ± 1.53	2.85 ± 2.31	0.27	0.80	0.69
*pfkmab*	Embryo	1.95 ± 1.56	3.16 ± 2.40	4.06 ± 2.26	4.05 ± 1.18	0.45	0.07	0.44	*pfkmab*	Embryo	2.67 ± 2.04	3.31 ± 2.33	2.07 ± 1.40	2.43 ± 1.52	0.52	0.34	0.86
	Alevin			3.99 ± 0.76	4.51 ± 0.92	0.19	**0**.**01**	0.59		Alevin			2.35 ± 1.48	3.01 ± 2.34	0.45	0.72	0.99
*pfkmba*	Embryo	1.33 ± 0.75	6.77 ± 5.33	4.03 ± 0.69	7.16 ± 5.87	0.02	0.35	0.49	*pfkmba*	Embryo	2.65 ± 1.87	7.94 ± 6.06	2.34 ± 1.74	4.86 ± 3.83	**0**.**02**	0.29	0.38
	Alevin			3.51 ± 1.06	6.82 ± 5.74	0.01	0.50	0.52		Alevin			3.38 ± 2.04	6.68 ± 5.92	**0**.**03**	0.88	0.59
*pfkmbb*	Embryo	2.40 ± 1.75	3.63 ± 2.69	5.53 ± 2.17	4.44 ± 0.95	0.93	**0**.**03**	0.17	*pfkmbb*	Embryo	4.34 ± 3.24	4.59 ± 3.52	3.20 ± 2.28	4.38 ± 3.05	0.58	0.59	0.71
	Alevin			4.84 ± 0.84	5.73 ± 1.51	0.17	**0**.**01**	0.82		Alevin			3.63 ± 2.45	4.46 ± 3.47	0.68	0.75	0.83
*pkmaa*	Embryo	2.30 ± 1.68	2.93 ± 2.13	5.66 ± 1.87	3.89 ± 1.02	0.43	**0**.**01**	0.10	*pkmaa*	Embryo	4.26 ± 3.00	3.93 ± 2.94	3.03 ± 2.15	3.29 ± 2.71	0.97	0.41	0.79
	Alevin			4.94 ± 0.55	4.96 ± 1.30	0.61	**0**.**00**	0.63		Alevin			3.49 ± 2.55	3.55 ± 2.94	0.91	0.63	0.87
*pkmab*	Embryo	3.48 ± 2.52	4.13 ± 3.30	7.48 ± 3.23	5.25 ± 1.21	0.48	**0**.**03**	0.21	*pkmab*	Embryo	4.74 ± 3.66	4.15 ± 3.22	3.77 ± 2.86	2.58 ± 1.62	0.47	0.30	0.81
	Alevin			6.96 ± 0.91	5.36 ± 0.90	0.60	**0**.**02**	0.22		Alevin			3.89 ± 2.59	3.35 ± 2.74	0.66	0.52	0.98
*pkmba*	Embryo	2.33 ± 1.78	3.19 ± 2.47	5.66 ± 1.85	4.39 ± 1.29	0.79	**0**.**01**	0.18	*pkmba*	Embryo	2.89 ± 2.07	3.28 ± 2.54	2.67 ± 1.98	2.3 ± 1.69	0.99	0.49	0.66
	Alevin			4.98 ± 0.45	4.24 ± 0.75	0.93	**0**.**01**	0.23		Alevin			2.75 ± 1.67	2.9 ± 2.25	0.77	0.77	0.89
*pkmbb*	Embryo	2.53 ± 1.71	3.66 ± 2.81	5.67 ± 2.45	4.99 ± 1.26	0.80	**0**.**02**	0.31	*pkmbb*	Embryo	3.45 ± 2.48	3.74 ± 2.87	3.07 ± 2.23	2.83 ± 2.01	0.98	0.52	0.79
	Alevin			5.58 ± 0.56	4,49 ± 0.79	0.98	**0**.**01**	0.13		Alevin			2.96 ± 1.69	3.44 ± 2.69	0.71	0.70	0.93
*pklr*	Embryo	3.78 ± 2.80	4.05 ± 2.93	7.61 ± 4.47	4.25 ± 1.11	0.23	0.12	0.16	*pklr*	Embryo	3.92 ± 2.84	3.67 ± 2.63	4.05 ± 3.38	2.69 ± 2.07	0.48	0.71	0.63
	Alevin			5.67 ± 1.14	4.74 ± 1.11	0.72	0.16	0.51		Alevin			3.72 ± 2.51	3.18 ± 2.68	0.72	0.76	0.90

N-NC, N-HC, H-NC and H-HC stands for fish subjected to normoxia and no carbohydrate diet, normoxia and high carbohydrate diet, hypoxia and no carbohydrate diet and hypoxia and high carbohydrate diet, respectively. Data were presented as mean ± SD (n = 6), statistical differences were evaluated by two-way *ANOVA* (*p* < 0.05, values in bold).

#### Genes involved in gluconeogenesis pathway

When fed the HC diet, trout exhibited a significant decrease in the mRNA levels of *pck1* and *fbp1b1*, and a significant increase in the mRNA level of *g6pcb2a* (Table 4, *p* ≤ 0.01). For the effect of hypoxia, trout subjected to the 2.5 mg·L^−1^ O_2_ hypoxic stimulus at either the embryo stage or the alevin stage displayed higher mRNA levels of *fbp1a* (*p* < 0.05) and *g6pcb2b* (*p* ≤ 0.01) than the normoxic group (Table 4). By contrast, the mRNA level of *g6pcb1a* was decreased in trout subjected to the 5.0 mg·L^−1^ O_2_ hypoxic stimulus at the embryo stage (Table 4, *p* = 0.05). Our results also highlighted a significant interaction between the diet received at first-feeding (*i*.*e*., HC or NC diet) and the level of oxygen during the early stimulus (*i*.*e*., normoxia *vs* hypoxia) for *g6pcb2b* in trout exposed to the 2.5 mg·L^−1^ O_2_ hypoxic stimulus at the embryo stage (Table 4, *p* ≤ 0.01).

**Table 4:** Effects of early hypoxia and high-carbohydrate diet at first-feeding on gene expressions involved in gluconeogenesis pathway.

Target gene	Hypoxia stage	Hypoxia level: 2.5 mg·L^−1^							Target gene	Hypoxia stage	Hypoxia level: 5.0 mg·L^−1^						
		N-NC	N-HC	H-NC	H-HC	*p* of Two-way *ANOVA*					N-NC	N-HC	H-NC	H-HC	*p* of Two-way *ANOVA*		
		Means ± SD	Means ± SD	Means ± SD	Means ± SD	Diet	Oxygen	D*O			Means ± SD	Means ± SD	Means ± SD	Means ± SD	Diet	Oxygen	D*O
*pck1*	Embryo	4.38 ± 2.63	1.46 ± 1.65	7.24 ± 6.04	1.25 ± 0.68	**0**.**00**	0.35	0.28	*pck1*	Embryo	5.54 ± 3.32	1.54 ± 1.87	3.60 ± 1.80	0.43 ± 0.23	**0**.**00**	0.09	0.64
	Alevin			6.73 ± 3.32	1.33 ± 0.79	**0**.**00**	0.25	0.20		Alevin			5.22 ± 5.05	1.03 ± 0.85	**0**.**01**	0.76	0.94
*pck2*	Embryo	2.13 ± 1.80	1.82 ± 1.29	3.73 ± 1.45	2.55 ± 0.79	0.20	0.05	0.45	*pck2*	Embryo	2.72 ± 1.60	2.56 ± 2.04	2.66 ± 2.76	2.52 ± 2.01	0.86	0.96	0.99
	Alevin			2.62 ± 0.40	2.62 ± 1.27	0.77	0.24	0.77		Alevin			3.82 ± 2.43	2.59 ± 2.38	0.43	0.52	0.55
*fbp1a*	Embryo	3.23 ± 2.48	3.04 ± 2.23	6.31 ± 2.59	4.17 ± 0.51	0.19	**0**.**02**	0.27	*fbp1a*	Embryo	4.07 ± 2.90	3.55 ± 2.59	3.53 ± 2.75	1.98 ± 1.15	0.31	0.30	0.61
	Alevin			5.83 ± 1.14	3.83 ± 0.57	0.15	**0**.**03**	0.23		Alevin			4.08 ± 2.82	2.91 ± 2.31	0.45	0.78	0.77
*fbp1b1*	Embryo	4.56 ± 3.30	2.06 ± 1.24	9.27 ± 5.58	3.14 ± 0.90	**0**.**01**	0.07	0.25	*fbp1b1*	Embryo	5.77 ± 4.37	2.11 ± 1.35	4.78 ± 3.82	1.94 ± 1.65	**0**.**02**	0.65	0.75
	Alevin			6.04 ± 1.64	3.31 ± 1.05	**0**.**00**	0.11	0.89		Alevin			4.73 ± 3.10	2.20 ± 1.96	**0**.**02**	0.70	0.64
*fbp1b2*	Embryo	2.96 ± 2.38	2.43 ± 1.71	4.93 ± 3.17	2.79 ± 0.51	0.15	0.20	0.38	*fbp1b2*	Embryo	3.83 ± 2.95	3.07 ± 2.19	3.48 ± 3.11	2.28 ± 1.53	0.35	0.59	0.83
	Alevin			4.25 ± 1.03	3.14 ± 0.61	0.22	0.14	0.66		Alevin			3.42 ± 2.27	2.51 ± 1.88	0.40	0.62	0.94
*g6pca*	Embryo	3.55 ± 3.00	3.05 ± 2.25	7.95 ± 5.58	4.02 ± 1.80	0.13	0.07	0.24	*g6pca*	Embryo	4.05 ± 3.02	2.81 ± 1.95	4.19 ± 3.17	2.20 ± 1.44	0.13	0.82	0.72
	Alevin			2.39 ± 1.78	4.54 ± 6.05	0.31	0.05	0.61		Alevin			3.77 ± 2.55	2.73 ± 2.43	0.28	0.87	0.92
*g6pcb1a*	Embryo	6.4 ± 6.81	8.61 ± 9.41	6.9 ± 2.26	6.38 ± 5.06	0.75	0.75	0.61	*g6pcb1a*	Embryo	6.60 ± 7.50	6.93 ± 7.21	2.77 ± 2.13	1.56 ± 1.38	0.84	**0**.**05**	0.73
	Alevin			8.96 ± 6.80	4.68 ± 2.91	0.72	0.81	0.26		Alevin			4.58 ± 3.69	5.58 ± 7.47	0.81	0.54	0.90
*g6pcb1b*	Embryo	2.98 ± 2.10	2.94 ± 1.87	6.24 ± 4.39	3.77 ± 0.86	0.26	0.07	0.27	*g6pcb1b*	Embryo	3.24 ± 2.22	2.77 ± 1.70	2.68 ± 2.18	2.42 ± 1.77	0.66	0.58	0.90
	Alevin			4.41 ± 1.00	3.94 ± 1.54	0.72	0.09	0.76		Alevin			2.96 ± 1.72	3.00 ± 2.68	0.81	0.97	0.77
*g6pcb2a*	Embryo	0.71 ± 0.90	3.23 ± 1.95	1.75 ± 1.15	3.32 ± 2.23	**0**.**01**	0.41	0.49	*g6pcb2a*	Embryo	0.54 ± 0.28	3.54 ± 2.08	1.10 ± 1.30	4.54 ± 5.72	**0**.**02**	0.55	0.86
	Alevin			1.22 ± 0.36	3.43 ± 2.32	**0**.**00**	0.59	0.81		Alevin			0.91 ± 0.71	4.99 ± 6.45	**0**.**02**	0.52	0.70
*g6pcb2b*	Embryo	1.75 ± 0.95	3.2 ± 2.20	5.30 ± 1.67	3.70 ± 1.38	0.91	**0**.**01**	**0**.**03**	*g6pcb2b*	Embryo	2.92 ± 1.94	3.97 ± 2.75	3.13 ± 2.47	3.02 ± 2.29	0.64	0.71	0.56
	Alevin			4.27 ± 0.45	4.52 ± 1.41	0.16	**0**.**00**	0.31		Alevin			2.92 ± 1.86	3.54 ± 2.77	0.40	0.83	0.83

N-NC, N-HC, H-NC and H-HC stands for fish subjected to normoxia and no carbohydrate diet, normoxia and high carbohydrate diet, hypoxia and no carbohydrate diet and hypoxia and high carbohydrate diet, respectively. Data were presented as mean ± SD (n = 6), statistical differences were evaluated by two-way *ANOVA* (*p* < 0.05, values in bold).

#### Genes involved in the glucose transport pathway

At first-feeding, the diet had no significant effect on the mRNA levels of glucose transport genes, with the exception of a significant increase in the *glut1ba* mRNA level (*p* ≤ 0.01) in trout subjected to the 2.5 mg·L^−1^ O_2_ hypoxic stimulus at the alevin stage (Table 5). However, compared with the normoxic group, the mRNA levels of *glut1aa*, *glu1ba* and *glut1bb* were significantly increased in trout subjected to the 2.5 mg·L^−1^ O_2_ hypoxic stimulus, regardless of the stage at which hypoxia was applied (*p* ≤ 0.01). Meanwhile, the mRNA level of *glut1ab* was significantly higher in trout subjected to 2.5 mg·L^−1^ O_2_ hypoxia at the alevin stage but not at the embryo stage (Table 5, (*p* < 0.05). By contrast, the *glut4a* mRNA level was increased in first-feeding trout subjected to this hypoxic stimulus at the embryo stage (*p* < 0.05) but not at the alevin stage (Table 5). No effect of the 5.0 mg·L^−1^ O_2_ hypoxic stimulus or of an interaction between the diet received at first-feeding (*i*.*e*., HC or NC) and the level of oxygen during the early stimulus (*i*.*e*., normoxia *vs* hypoxia) was found on the mRNA levels of genes involved in glucose transport (Table 5).

**Table 5:** Effects of early hypoxia and high-carbohydrate diet at first-feeding on gene expressions involved in glucose transport pathway.

Target gene	Hypoxia stage	Hypoxia level: 2.5 mg·L^−1^							Target gene	Hypoxia stage	Hypoxia level: 5.0 mg·L^−1^						
		N-NC	N-HC	H-NC	H-HC	*p* of Two-way *ANOVA*					N-NC	N-HC	H-NC	H-HC	*p* of Two-way *ANOVA*		
		Means ± SD	Means ± SD	Means ± SD	Means ± SD	Diet	Oxygen	D*O			Means ± SD	Means ± SD	Means ± SD	Means ± SD	Diet	Oxygen	D*O
*glut1aa*	Embryo	2.12 ± 2.04	0.45 ± 0.84	5.54 ± 5.89	10.54 ± 6.80	0.39	**0**.**00**	0.09	*glut1aa*	Embryo	3.10 ± 4.26	0.36 ± 0.77	3.94 ± 4.59	4.31 ± 7.53	0.56	0.25	0.45
	Alevin			9.55 ± 5.03	6.86 ± 3.35	0.11	**0**.**00**	0.70		Alevin			4.88 ± 6.11	1.82 ± 3.46	0.10	0.35	0.93
*glut1ab*	Embryo	2.60 ± 2.08	3.54 ± 3.02	5.65 ± 2.37	4.37 ± 1.35	0.86	0.05	0.25	*glut1ab*	Embryo	3.97 ± 2.98	3.89 ± 3.18	2.99 ± 2.04	3.07 ± 2.57	1.00	0.43	0.94
	Alevin			5.21 ± 0.69	4.63 ± 0.96	0.82	**0**.**03**	0.34		Alevin			3.87 ± 2.86	2.79 ± 2.18	0.62	0.61	0.67
*glut1ba*	Embryo	2.38 ± 1.57	4.64 ± 3.57	6.24 ± 2.20	7.36 ± 1.70	0.10	**0**.**00**	0.57	*glut1ba*	Embryo	3.66 ± 2.78	4.99 ± 3.75	3.09 ± 2.35	3.46 ± 2.34	0.47	0.38	0.68
	Alevin			5.26 ± 0.78	7.70 ± 1.41	**0**.**01**	**0**.**00**	0.92		Alevin			3.29 ± 2.23	3.93 ± 2.87	0.42	0.56	0.78
*glut1bb*	Embryo	2.45 ± 1.56	4.03 ± 2.92	5.43 ± 2.13	5.53 ± 0.86	0.32	**0**.**01**	0.38	*glut1bb*	Embryo	3.55 ± 2.47	4.52 ± 3.17	3.27 ± 2.41	3.23 ± 2.32	0.67	0.47	0.64
	Alevin			5.15 ± 1.08	5.44 ± 0.70	0.21	**0**.**01**	0.38		Alevin			3.35 ± 2.32	3.80 ± 2.86	0.53	0.68	0.82
*glut2a*	Embryo	2.59 ± 1.90	3.40 ± 2.61	5.05 ± 2.86	4.29 ± 1.69	0.98	0.09	0.42	*glut2a*	Embryo	3.13 ± 2.34	4.11 ± 3.11	3.23 ± 2.62	2.29 ± 1.59	0.99	0.40	0.36
	Alevin			3.28 ± 0.96	3.86 ± 0.99	0.35	0.43	0.87		Alevin			2.76 ± 2.32	3.25 ± 2.60	0.50	0.57	0.82
*glut2b*	Embryo	2.22 ± 1.68	3.32 ± 2.28	6.06 ± 4.54	3.78 ± 0.93	0.60	0.07	0.14	*glut2b*	Embryo	2.80 ± 2.14	3.40 ± 2.26	2.83 ± 2.41	2.14 ± 1.23	0.96	0.48	0.45
	Alevin			3.86 ± 1.05	4.01 ± 1.55	0.38	0.11	0.60		Alevin			2.86 ± 2.03	3.08 ± 2.63	0.66	0.89	0.84
*glut4a*	Embryo	2.59 ± 1.75	3.76 ± 2.72	5.45 ± 1.69	4.77 ± 1.47	0.77	**0**.**03**	0.27	*glut4a*	Embryo	3.48 ± 2.37	3.77 ± 2.64	2.63 ± 1.78	2.64 ± 1.79	0.87	0.28	0.88
	Alevin			5.03 ± 0.99	4.20 ± 0.71	0.82	0.06	0.17		Alevin			2.99 ± 1.95	2.88 ± 2.08	0.93	0.47	0.83
*glut4b*	Embryo	3.15 ± 2.36	4.43 ± 3.13	5.49 ± 1.26	5.60 ± 1.26	0.44	0.06	0.51	*glut4b*	Embryo	3.57 ± 2.51	4.29 ± 3.13	2.48 ± 1.92	2.84 ± 2.03	0.59	0.22	0.86
	Alevin			5.76 ± 1.10	4.96 ± 0.57	0.78	0.08	0.23		Alevin			2.84 ± 1.90	3.00 ± 2.21	0.67	0.33	0.78

N-NC, N-HC, H-NC and H-HC stands for fish subjected to normoxia and no carbohydrate diet, normoxia and high carbohydrate diet, hypoxia and no carbohydrate diet and hypoxia and high carbohydrate diet, respectively. Data were presented as mean ± SD (n = 6), statistical differences were evaluated by two-way *ANOVA* (*p* < 0.05, values in bold).

## Discussion

Rainbow trout belongs to a high trophic level, defining it as a carnivorous fish that is metabolically adapted for the high catabolism of proteins and the low use of dietary carbohydrates^35, 36^. The latter physiological feature is considered a constraint in the search for alternative feedstuffs to minimise the dependence on fish meal by increasing the carbohydrate content in the diet of trout. In this context, early nutritional programming has emerged as a new nutritional strategy to improve the use of dietary carbohydrates in carnivorous fish while avoiding the need to set up large-scale genetic breeding programmes or to genetically modify the animals^11, 12^. The exploration of this strategy is only in its beginnings in trout^11, 12^ and requires deeper investigations to be optimised. The present study is the first to test the programming effect of a non-nutritional stimulus, hypoxia applied during early developmental stages on glucose metabolic pathways in trout at first-feeding. A 24 h acute hypoxic stimulus (2.5 or 5.0 mg·L^−1^ O_2_) was applied to two different stages: non-hatched embryos (152 °D) and early hatched alevins (552 °D), which were long before the first-feeding (654 °D).

To test whether the hypoxic stimulus had a physiological impact on the fish, we first analysed the mRNA level of genes that are known to mediate the hypoxic response of cells. We observed a dramatic increase in mRNA levels of *egln3a* and *egln3b* genes in hypoxia-treated groups compared with the normoxic controls. Genes encoding the Egl-9 homologue 3 protein are considered as cellular oxygen sensors whose transcription is induced by hypoxia *via* the hypoxia-inducible factor 1 (Hif1)-meditated pathway^37, 38^. The induction of the expression of these duplicated genes confirmed the physiological response of both embryos and alevins to the hypoxic stimulus. However, no significant difference among treatments was found in the mRNA levels of Hif1 encoding genes, the master regulators of the cellular hypoxia response. This result was not completely surprising because *hif1* is reported to be regulated predominantly at the post-translational level^39^.

Limited oxygen availability is also known to drive a switch in metabolism from mitochondrial aerobic oxidative phosphorylation to an anaerobic glycolytic pathway^16^, resulting in increased lactate production^23, 26^ and glucose uptake^21, 40, 41^. In the present study, the higher mRNA level of the glucose transporter *glut1ba* as well as the up-regulation of one of the duplicated proton-lactate symporter encoding genes (*slc16a3a*) in fish subjected to hypoxia suggests the formation of a functional cellular response to hypoxia at the metabolic level. Indeed, similar to *egln3a* and *egln3b*, *glut1 and slc16a3* were also reported to be downstream target genes of Hif1^22, 42^. Together, our results confirm that the hypoxic stimulus was being sensed by both embryos and alevins at the cellular level.

**Figure 2:**
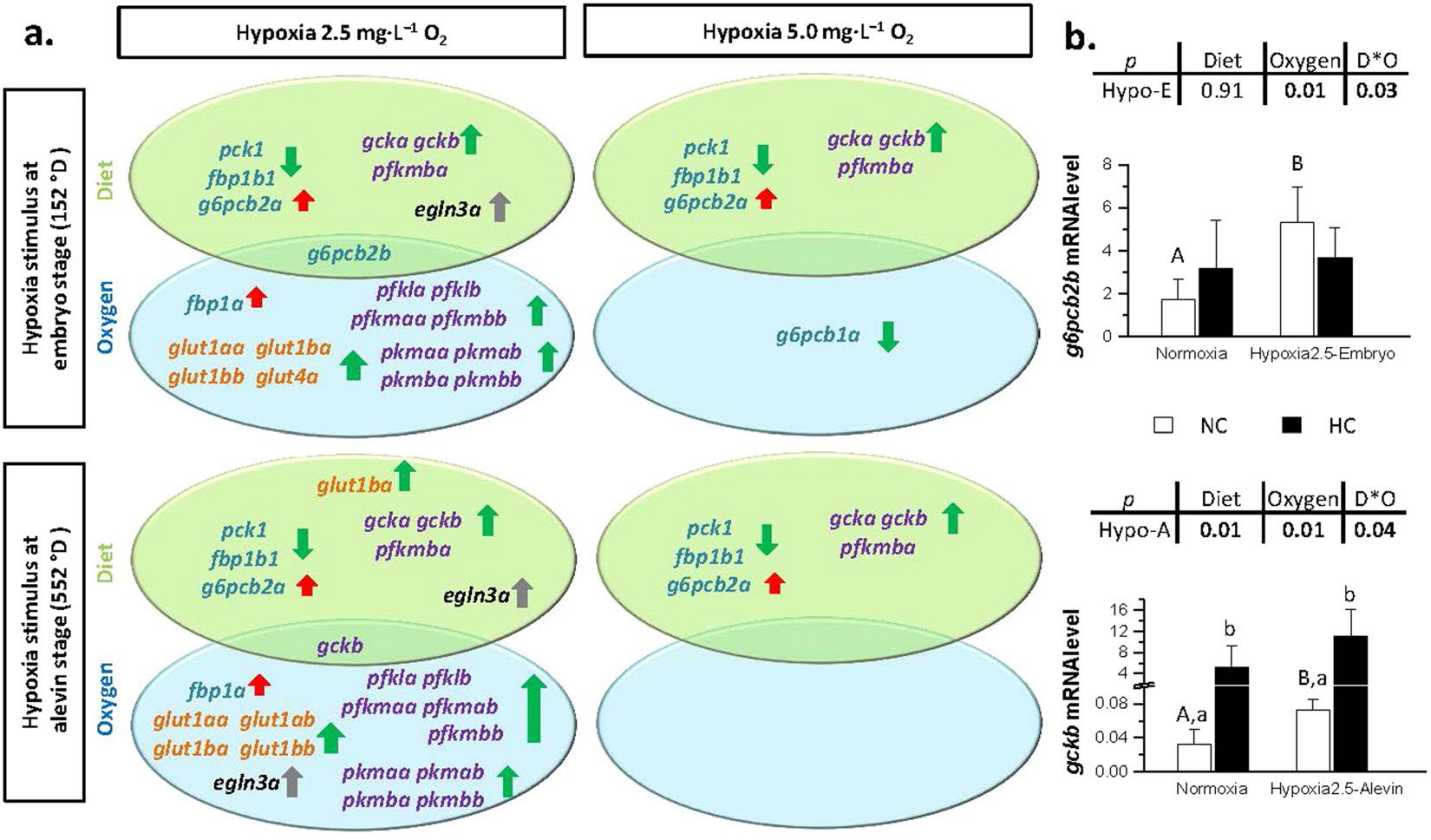
An overview of the effects of oxygen and diet on the mRNA levels of glucose metabolism-related genes and *egln3* genes in trout at first-feeding. (**a**) mRNA levels of genes significantly affected by high carbohydrate diet (green oval) or by the hypoxic stimulus (blue oval) at first-feeding. Genes written in blue, purple and orange belong to gluconeogenic, glycolytic and glucose transport pathway, respectively. Arrows represent up- or down- modulation of the mRNA level, with green colour in favour of glucose expenditures and red colour in favour of glucose production. (**b**) Effect of interactions between the early hypoxic stimulus and the high carbohydrate diet on mRNA levels of *g6pcb2b* and *gckb* in trout at first-feeding. White and black bars stand for trout fed the no carbohydrate (NC) or the high carbohydrate (HC) diet at first-feeding, respectively. Hypo-E and Hypo-A stands for trout subjected to hypoxic stimulus at embryo or alevin stage, respectively. Data are presented as the mean ± SD (n = 6), and significant differences were evaluated by two-way *ANOVA* followed by a subsequent one-way *ANOVA* analysis (*p* < 0.05). Capital and lowercase letters indicate the significant effect (*p* < 0.05) of early hypoxic stimulus or high carbohydrate diet within their corresponding groups, respectively.

We then investigated the combined effects of early hypoxic stimulus and a high carbohydrate diet on the mRNA levels of glucose-metabolism related genes in trout alevins at first-feeding. A summary of these effects is given in Fig. 2. Note that, the gene expression assays were conducted in the whole body of embryos or alevins because of their small size limited their dissection.

The effects of a high dietary carbohydrate supply at first-feeding on glucose metabolism-related genes were similar to a previous report by Marandel *et al*.^15^ in trout. These effects include the increase in mRNA levels of the glycolytic genes *gcka*, *gckb*, the inhibition of the gluconeogenic genes *pck1* and *fbp1b1*, and also an atypical up-regulation of *g6pcb2a* (Fig. 2a). In addition, our results showed an increase in the mRNA level of *pfkmba* in trout fed the HC diet at first-feeding, one of the four *pfkm* paralogous genes involved in the muscular glycolysis pathway. It is the first time that different paralogous genes of *pfkm* have been identified and separately amplified. This increase in the *pfkm* mRNA level in trout fed a high carbohydrate diet was never detected in studies published before the sequencing of the trout genome^34^. These studies investigated only one gene called 6PFK-M^12, 43^. Such a difference in mRNA level response between the four paralogous genes suggests a neo- or a sub-functionalisation of *pfkmba* in relation to dietary glucose. Regarding the glucose transport genes in general, there was no significant effect of the diet, confirming the results obtained in first-feeding alevins in a previous nutritional programming study^12^.

Our results showed a clear programming effect of the early acute hypoxic stimulus on glucose metabolism-related genes at first-feeding, either directly or interacting with the dietary carbohydrate content received at first-feeding (Fig. 2a).

With respect to the effects of hypoxia, we observed that the moderate hypoxic stimulus (5.0 mg·L^−1^ O_2_) did not induce a durable modification of the glucose metabolism in trout at the time of first-feeding (except for a downregulation of *g6pcb1a* in trout exposed to this level of hypoxia at the embryo stage). By contrast, the more severe level of hypoxia (2.5 mg·L^−1^ O_2_) increased the mRNA levels of genes involved in glycolysis (*pfkla*, *pfklb*, *pfkmaa*, *pfkmbb*, *pkmaa*, *pkmab*, *pkmba*, *pkmbb*), gluconeogenesis (*fbp1a*, *g6pcb2b*) and glucose transport (*glut1aa*, *glut1ba*, *glut1bb*), irrespective of the time at which the stimulus was applied. The overall higher mRNA levels of glycolytic genes and of *glut1* paralogous genes in the hypoxic treatment group is in line with previous observations in vertebrates, including fish^44–46^. It is well established that under hypoxic conditions, the uptake of glucose and the glycolytic flux are cooperatively induced to ensure enough ATP production to compensate for the lower efficiency of glycolysis compared with oxidative phosphorylation. These compensations are mainly achieved by upregulating genes encoding for glucose transporters (especially *glut1* and *glut3*)^21, 47^ and glycolytic enzymes^16, 24, 25^ in a Hif-1 dependent manner. Meanwhile, the increase in the mRNA levels of gluconeogenetic *fbp1b1* and *g6pcb2b* in fish subjected to 2.5 mg·L^−1^ O_2_ hypoxia seems paradoxical, but such a phenomenon has also been reported in previous studies^48–50^. A few genes were also differentially affected depending on the developmental stage at which they received the hypoxic stimulus (*i*.*e*., embryo or alevin). Indeed, *gckb*, *pfkmab*, and *glut1ba* mRNA levels were altered by the hypoxic stimulus when applied in alevins, whereas the *glut4a* mRNA level was increased only in trout subjected to hypoxia at the embryo stage.

Finally, it should be noted that the higher mortality rate observed before first-feeding in the fish subjected to the 2.5 mg·L^−1^ O_2_ hypoxia at the alevin stage did not seem to have selected animals who were better metabolically adapted (at least concerning glucose metabolism) to hypoxia because the effects of hypoxia were similar to those in fish subjected to hypoxia at the embryo stage (with no differences in survival). Both stages (152 °D and 552 °D) thus appeared to be good “windows of metabolic plasticity”. Indeed, an early acute hypoxic stimulus (2.5 mg·L^−1^ O_2_) applied at the embryo stage or the alevin stage succeeded in inducing similar programming effects on glucose metabolism in rainbow trout at first-feeding, at least on the mRNA level.

Interestingly, at the time of first-feeding, the mRNA level of *egln3a* remained significantly higher in trout which had experienced 2.5 mg·L^−1^ O_2_ hypoxia at the alevin stage (552 °D) despite their normoxic rearing conditions. Similar results have been found in European sea bass, in which an early hypoxia exposure during the larval stage induced a long-term overexpression of *egln3* in juveniles^51^. The authors hypothesised that transcriptional imprinting occurs during early exposure to hypoxia and should lead to the enhancement of the Hif-1α stabilisation and thus the modulation of *egln3*, known to be regulated in a Hif-dependent manner^37, 52, 53^. Moreover, the mRNA level of *egln3a* was significantly affected by the diet at first-feeding in fish exposed to the early 2.5 mg·L^−1^ O_2_ hypoxic stimulus, either at the embryo stage or the alevin stage, as well as the normoxic group. This upregulation may also be attributed to the stabilisation of Hif-1α, but this time possibly meditated by the FIH-1 activity, which was induced by the excessive ROS produced in a high glucose environment^54^.

Even more noteworthy, interactive effects of the early hypoxic stimulus and the diet received at first-feeding were observed on the mRNA level of *g6pcb2b* and *gckb* in trout exposed to 2.5 mg·L^−1^ O_2_ hypoxia at the embryo stage and the alevin stage, respectively. Indeed, in the normoxic group, *g6pcb2b* and *gckb* mRNA levels were both up-regulated in trout fed the HC diet; a completely reverse pattern for *g6pcb2b* or an intensified effect for *gckb* were observed in fish exposed to 2.5 mg·L^−1^ O_2_ hypoxia at the embryo and alevin stages, respectively (Fig. 2b). These results are in favour of an inhibition of the last step of gluconeogenesis and an increase in the first step of glycolysis, indicating that an early hypoxic stimulus may modify the glucose utilisation at first-feeding in trout to some extent by affecting *g6pcb2b* expression, considered to be involved in the glucose-intolerant phenotype in trout juveniles^14^.

In summary, the present study confirms the possibility of modulating glucose metabolism-related genes in trout by using a nutritional programming strategy during early development with a non-nutritional stimulus: hypoxia. Further investigations are now required to explore whether there is a long-term programming effect of an early hypoxic stimulus alone or combined with a high carbohydrate diet at first-feeding on glucose metabolism in trout at later life stages.

## Methods

### Ethical issues and approval

Investigations were conducted according to the guiding principles for the use and care of laboratory animals and in compliance with French and European regulations on animal welfare (Décret 2001-464, 29 May 2001 and Directive 2010/63/EU, respectively). This protocol and the project as a whole were approved by the French National Consultative Ethics Committee (reference numbers 2015112018112159 and 201511201756973).

### Experimental design and diets

#### Diets

Two isolipidic and isoenergetic experimental diets containing either no carbohydrates (NC, 0% carbohydrates) or a high content of digestible carbohydrates (HC, ~60% carbohydrates) were prepared in our own facilities (INRA, Donzacq, France) as extruded pellets (see Supplementary Table S2). Glucose and gelatinised starch were included as the carbohydrate sources, fish meal was used as protein source, and dietary lipids were provided by fish oil and fish meal. The large increase in dietary carbohydrate content in the HC diet was compensated for by a decrease in protein content.

#### Fish and Experimental design

**Figure 3:**
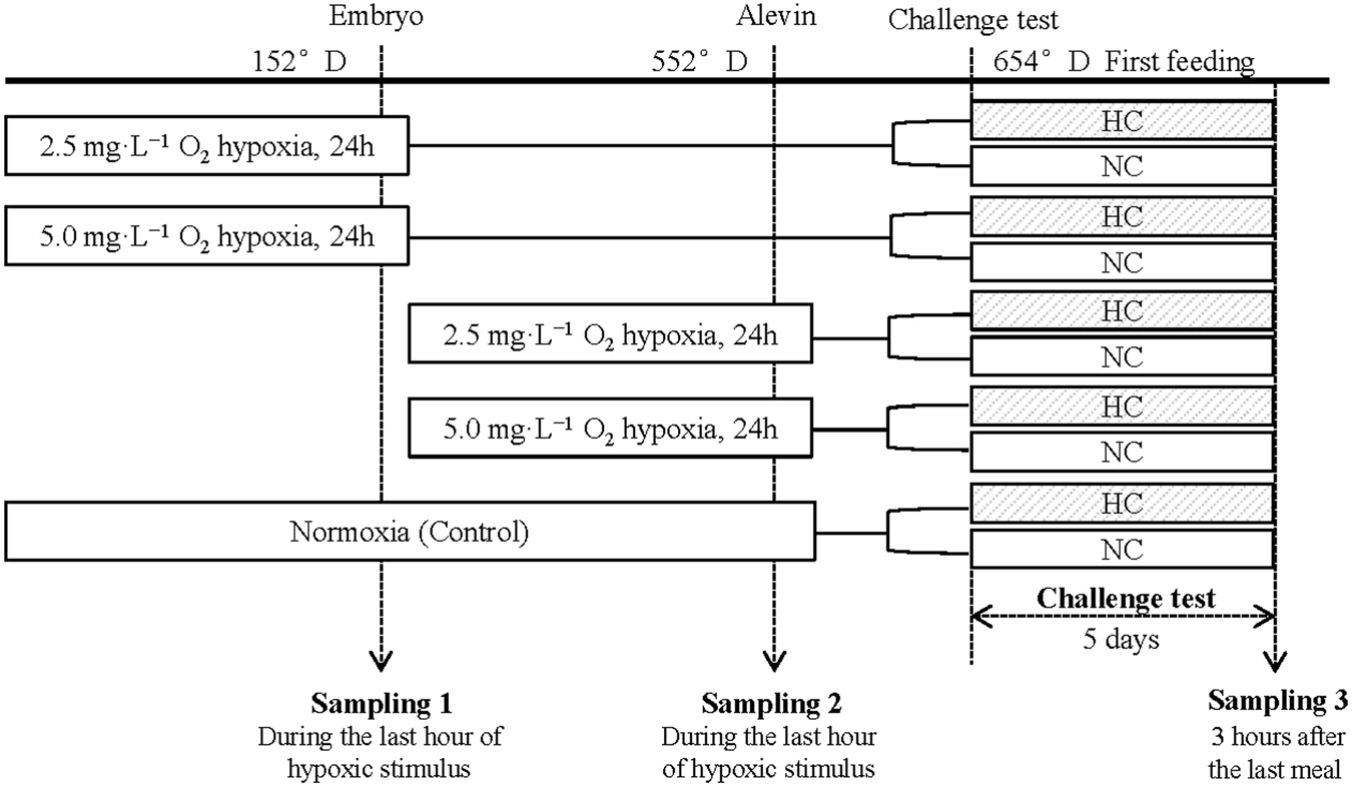
Experimental design. Two levels of hypoxic stimulus (2.5 or 5.0 mg·L^−1^ O_2_) were applied to rainbow trout for 24 h at the embryo (152 °D) or alevin stages (552 °D). The fish were then fed either a high carbohydrate (HC) or a no carbohydrate (NC) diet at first-feeding. The normoxic group serves as the control.

A pool of oocytes was fertilised with neomale (the fish with a male phenotype but genetically female) sperm and reared in 30 separate tanks at 8 °C in the INRA experimental facilities, Lees-Athas, France. A 24 h-acute hypoxic stimulus (2.5 mg·L^−1^ or 5.0 mg·L^−1^ dissolved oxygen) was applied to embryos at the primitive liver setting-up stage (stage 21 based on the Vernier table) at 152 °D or on alevins at 552 °D just after hatching and before the first-feeding^55^. Two different levels of hypoxia were used at each stage: 2.5 mg·L^−1^ or 5.0 mg·L^−1^ of dissolved oxygen. Levels of hypoxia were obtained by bubbling nitrogen in the water in which the tanks containing the fish were immersed. Levels of hypoxia were checked every two hours using an oximeter (Hach, Germany). After the stimulus, fish were put back in normoxic conditions. Fish kept continuously in a normoxic conditions (11.0 mg·L^−1^ dissolved oxygen) were used as a control. Samples were taken during the last hour of the hypoxic stimulus (Fig. 3, 30 embryos per tank in sampling1 and 1 alevin per tank in sampling 2). For 5 days from the first-feeding meal, each group of fish was fed with either the HC or NC diets and 2 fish per tank were sampled 3 h after the last meal (Fig. 3, sampling 3; corresponding to the postprandial peak in juveniles). The 30 tanks consisted of 10 treatments in total, with 6 replicated tanks per treatment in sampling 1 and sampling 2 and triplicated tanks per treatment in sampling 3 (Fig. 3). Embryos were directly snap-frozen in liquid nitrogen, whereas alevins were killed by terminal anaesthetisation by bathing in benzocaine (30 mg·L^−1^) prior to sampling and subsequently frozen in liquid nitrogen. Samples were stored at −80 °C until further analysis.

### Analytical methods

The chemical composition of the diets was analysed according to the following procedures: dry matter was determined after drying to constant weight at 105 °C; crude protein (N × 6.25) was determined by the Kjeldahl method after acid digestion; crude lipid was determined by petroleum ether extraction (Soxtherm); gross energy was measured in an adiabatic bomb calorimeter (IKA, Heitersheim Gribeimer, Germany); ash was estimated through incinerating in a muffle furnace for 6 h at 600 °C.

### *In silico* analysis

Orthologous genes of mammalian *Hif1α*, *Pkm*, *Pdk1*, *Ldha*, *Slc16a3*, *Slc2a1* (also called *Glut1*), *Slc2a2* (also called *Glut2*), *Slc2a4* (also called *Glut4*), *Pfkm* and *Egln3* in the rainbow trout genome^34^ were identified in the *Oncorhynchus mykiss* genome browser (Genoscope: http://www.genoscope.cns.fr/trout/) and extracted from the SIGENAE database (http://www.sigenae.org) using the BLAST tool (all accession numbers are given in Supplementary Table S1). Sequences from other species were collected from the Ensembl Genome database (Ensembl release 84, March 2016: http://www.ensembl.org). Genomicus software, v01.01 (http://www.genomicus.biologie.ens.fr/genomicus-trout-01.01/cgi-bin/search.pl) was used to confirm the identities of the genes cited above by syntenic analysis (see Supplementary File). When the syntenic analysis was not sufficient to confirm the identity/annotation of the genes a phylogenetic analysis was performed using the MEGA package V6 software^56^ as previously described^14^. The phylogenetic tree, based on deduced full-length amino acid sequences, was produced using the neighbour-joining (NJ) method and confirmed by the minimum evolution method (see Supplementary File). The reliability of the inferred trees was estimated using the bootstrap method with 1000 replications. New gene annotations were allocated according to ZFIN Nomenclature guidelines (http://zfin.org/, Supplementary Table S1).

### Total RNA extraction and cDNA synthesis

Total RNA extraction was conducted on the whole body of embryos or alevins. Samples were homogenised in Trizol reagent (Invitrogen) with Precellys®24 (Bertin Technologies, Montigny-le-Bretonneux, France) according to the process described previously^15^. Luciferase control RNA (Promega), 10 pg per 1.9 mg of embryo/alevin was added to each sample to allow for data normalisation as previously described^57, 58^. Total RNA was then extracted according to the Trizol manufacturer’s instructions and 1 µg of total RNA was subsequently reverse-transcribed to cDNA using The Super-Script III RNAse H-Reverse transcriptase kit (Invitrogen) with random primers (Promega, Charbonniéres, France).

### Quantitative real-time-PCR

The primer sets used for analysis are listed in Supplementary Table S1. The newly designed primer sets used in quantitative real-time-PCR (qPCR) assays for hypoxic response and glucose metabolism-related genes were validated on a pool of cDNA and the amplified products were sequenced systematically. The primers that amplified *gck*, *pfkl*, *pkl* genes and gluconeogenic genes, which had already been published in previous studies^14, 15^, are not shown in Supplementary Table S1. QPCR assays were performed with the Roche Lightcycler 480 system (Roche Diagnostics, Neuilly-sur-Seine, France). The reaction mix was 6 µl per sample, including 2 µl of diluted cDNA template (1:25), 0.12 µl of each primer (10 µmol l^−1^), 3 µl of Light Cycler 480 SYBR® Green I Master mix and 0.76 µl of DNAse/RNAse-free water (5 Prime GmbH, Hamburg, Germany). The qPCR protocol was initiated at 95 °C for 10 min for the initial denaturation of the cDNA and hot-start Taq-polymerase activation, followed by 45 cycles of a two-step amplification programme (15 s at 95 °C; 10 s at 60 °C). Melting curves were monitored systematically (temperature gradient 0.11 °C per second from 65 to 97 °C) at the end of the last amplification cycle to confirm the specificity of the amplification reaction. Each qPCR assay included replicate samples (duplicate of reverse transcription and PCR amplification) and negative controls (reverse transcriptase- and cDNA template-free samples). Data were subsequently normalised to the exogenous luciferase transcript abundance in samples diluted at 1:25 using the E method (Light Cycler software) as previously described^58^.

### Statistical analysis

Zootechnical data (*i*.*e*., survival and malformation) were analysed by a Pearson’s Chi-squared test. For qPCR results of hypoxic responsive genes (*i*.*e*., results obtained from sampling 1 and sampling 2, Fig. 3), normality of distributions was assessed by Shapiro-Wilk test. Data were then analysed by the Kruskal-Wallis non-parametric test followed by Tukey’s test as the *post hoc* analysis. qPCR results concerning the *egln3* genes and glucose metabolism-related genes (from sampling 3, Tables 2, 3, 4 and 5) were analysed by a two-way *ANOVA* with oxygen and diet as independent variables. When oxygen x diet interaction was significant, means were compared using one-way *ANOVA*. Differences were considered statistically significant at *p* < 0.05. Data were analysed using the R software (v3.1.0)/R Commander Package.

## Electronic supplementary material

Supplemental materials
