# Supplementary material for: Exposure to an acute hypoxic stimulus during early life affects the expression of glucose metabolism-related genes at first-feeding in trout
Source: Sci Rep. 2017 Mar 23;7:363. doi: 10.1038/s41598-017-00458-4 (PMC5428409; doi:10.1038/s41598-017-00458-4)
Supplement: Supplementary file 1 — Supplemental materials [file 41598_2017_458_MOESM1_ESM.doc]

**Exposure to an acute hypoxic stimulus during early life affects the expression of glucose metabolism-related genes at first-feeding in trout**

Jingwei Liu, Elisabeth Plagnes-Juan, Inge Geurden, Stéphane Panserat, Lucie Marandel^§^

**Supplementary Information**

*In silico* analysis

***hif1α***

Two sequences related to *Hif1α* were found in the rainbow trout genome and seemed to group with the *hif1αb* genes of the zebrafish and the cave fish (Fig. S1). They were also included in a syntenic group conserved between mammals and teleosts (Fig. S2). To confirmed their identity, an identity matrix was calculated based on amino acid sequences which demonstrated that the two sequences in trout shared both around 68% of identity with the zebrafish Hifα1b against around 55% with the zebrafish Hifα1a (data not shown). We thus annotated them as *hifα1b1* (scaffold 1551, GSONMG00056387001) and *hifα1b2* (scaffold 3, GSONMG00076681001) and suggested that the two *hifα1a* ohnologs should have been lost after the salmonid-specific 4th whole genome duplication (Ss4R).


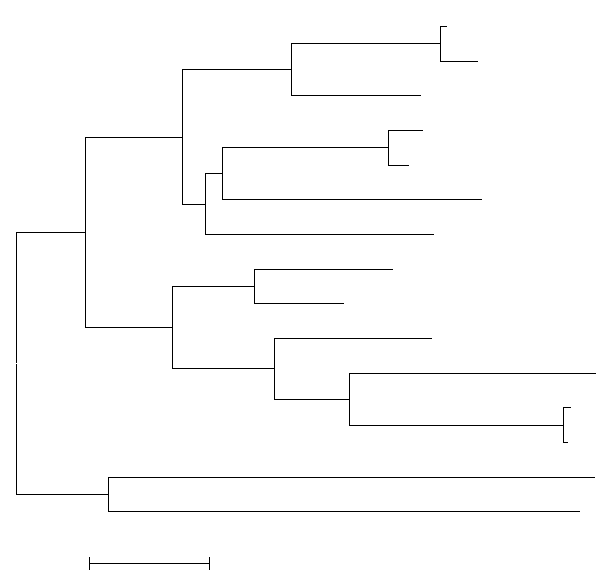


*Tetraodon nigroviridis* (ENSTNIT00000020708)

*Tetraodon nigroviridis* (ENSTNIT00000020709)

*Takifugu rubripes* (ENSTRUT00000030723)

100

*Oncorhynchus mykiss* (GSONMG00056387001) scaffold_1551-*hif1αb1*

*Oncorhynchus mykiss* (GSONMG00076681001) scaffold_3-*hif1αb2*

*Gasterosteus aculeatus* (ENSGACT00000011293)

*Poecilia formosa* (ENSPFOT00000008152)

*Astyanax mexicanus (*ENSAMXT00000019920) *hif1αb*

*Danio rerio* (ENSDART00000018500) *hif1*α*b*

*Lepisosteus oculatus* (ENSLOCT00000012442)

*Xenopus tropicalis* (ENSXETT00000031610)

*Mus musculus* (ENSMUST00000021530)

*Homo sapiens* (ENST00000539097)

*Danio rerio (*ENSDART00000044282) *hif1αa*

*Astyanax mexicanus (*ENSAMXT00000019920) *hif1αa*

99

100

96

51

52

99

94

98

92

100

99

0.05

**Fig. S1 Pylogenetic of *hif1a* genes.** The phylogenetic trees were built by the neighbor-joining (NJ) method. The reliability of the inferred trees was estimated by the bootstrap method with 1,000 replications. All accession numbers (from GenBank, Ensembl, or Genoscope databases) are specified in parentheses.


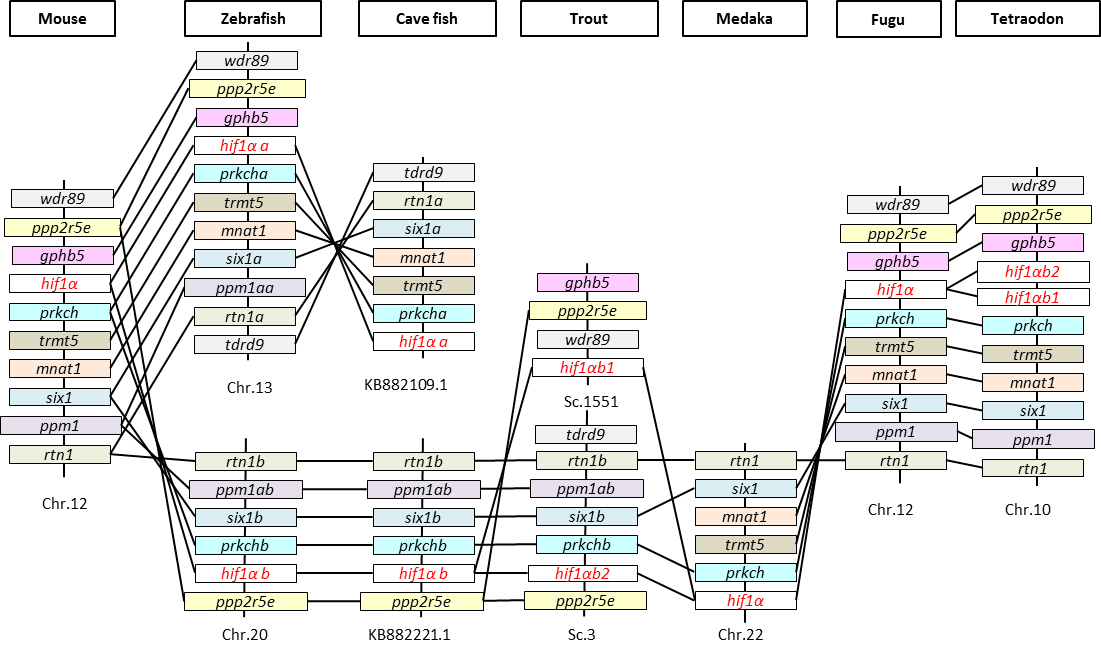


**Fig. S2 Syntenic analysis of *hif1a* genes.** Conserved synteny around the *hif1α* loci in mammals and teleosts. Data were collected with Genomicus software version 01.01, and *hif1α* genes were annotated by ourselves according to our phylogenetic analysis following ZFIN nomenclature guidelines. Chr., chromosome; sc., scaffold.

***ldha, egln3, slc2a4***


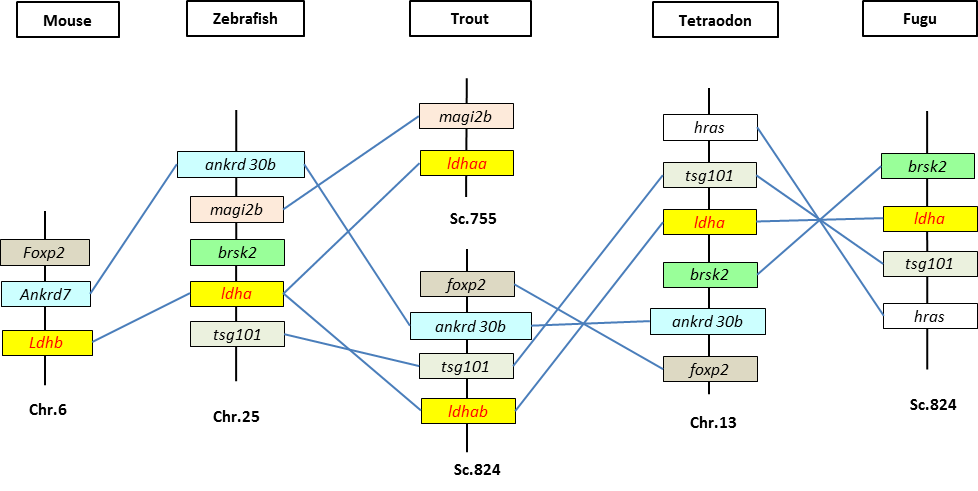
 For *Ldha*, *Egln3* and *Slc2a4* our analysis revealed that only one gene was conserved in the zebarfish, medaka, tetraodon and fugu genomes demonstrating that after the teleost-specific whole-genome duplication (Ts3R) one copy of each of these genes was lost. In trout, two sequences related to each of these genes were found and our syntenic analysis showed that both copies were located on different scaffolds but included in syntenic groups conserved between mammals and teleosts. These findings suggested that after the Ss4R, a pair of ohnologous genes were conserved in the trout genome for *ldha* (Fig. S3), *egln3* (Fig. S4) and *slc2a4* (Fig.S5). We annotated them arbitrarily « a » and « b » as mentionned in the Table 2.

**Fig. S3 Syntenic analysis of *ldha* genes.** Conserved synteny around the *ldha* loci in mammals and teleosts. Data were collected with Genomicus software version 01.01, and *ldha* genes were annotated by ourselves according to our syntenic analysis following ZFIN nomenclature guidelines. Chr., chromosome; sc., scaffold.


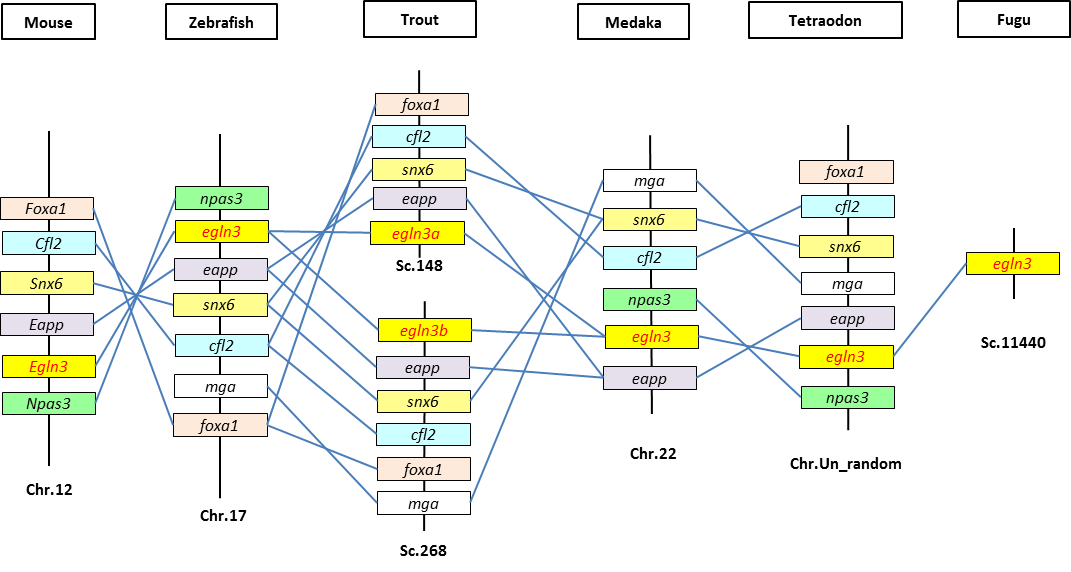

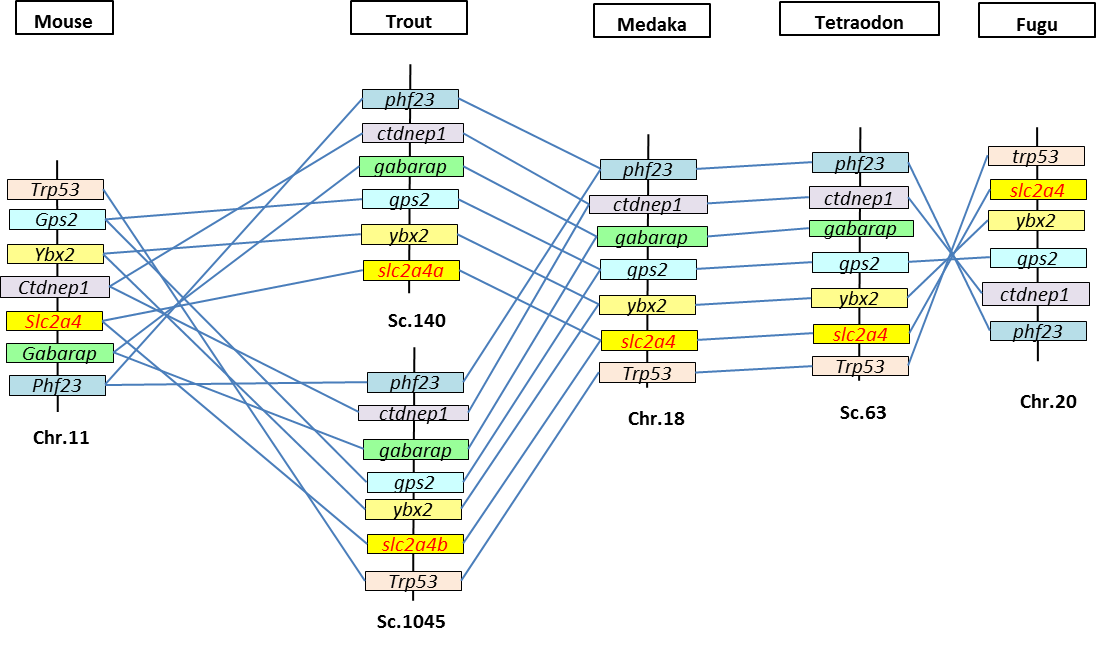
**Fig. S4 Syntenic analysis of *egln3* genes.** Conserved synteny around the *egln3* loci in mammals and teleosts. Data were collected with Genomicus software version 01.01, and *egln3* genes were annotated by ourselves according to our syntenic analysis following ZFIN nomenclature guidelines. Chr., chromosome; sc., scaffold.

**Fig. S5 Syntenic analysis of *slc2a4* genes.** Conserved synteny around the *slc2a4* loci in mammals and teleosts. Data were collected with Genomicus software version 01.01, and *slc2a4* genes were annotated by ourselves according to our syntenic analysis following ZFIN nomenclature guidelines. Chr., chromosome; sc., scaffold

***pdk1***

The analysis of the rainbow trout genome showed that only one gene related to the mammalian *Pdk1* existed in this species as well as in other teleosts. This gene was located on scaffold 18 (GSONMG00069580001) in the *cir1-sp9-sp3-hat1-dlx1a-itga6a* syntenic group conserved in the mouse, zebrafish, medaka, fugu and tetraodon around the *pdk1* gene. This syntenic group was found duplicated on scaffold 26 but did not contain any ohnologous *pdk1* gene. This last finding indicated that one copy of *pdk1* gene was lost after the Ss4R in the rainbow trout genome (Fig. S6).


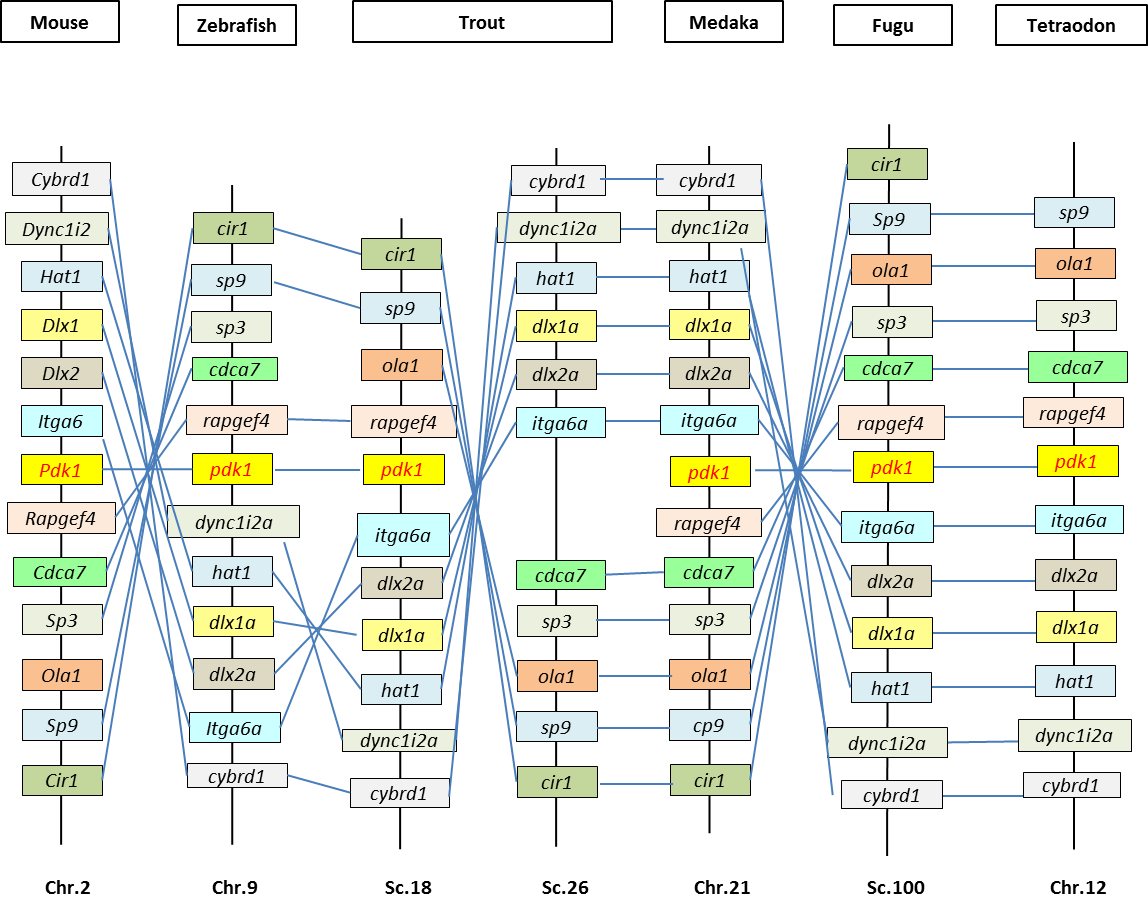


**Fig. S6 Syntenic analysis of *pdk1* genes.** Conserved synteny around the *pdk1* loci in mammals and teleosts. Data were collected with Genomicus software version 01.01, and *pdk1* genes were annotated by ourselves according to our syntenic analysis following ZFIN nomenclature guidelines. Chr., chromosome; sc., scaffold.

***slc16a3***

Then, we found two sequences related to Slc16a3 in the rainbow trout genome. However, the syntenic analysis was not relevant as no conserved syntenic group had been identified probably because the scaffolds bearing the genes were too short to allow this identification. We performed a phylogenetic analysis which demonstrated that the rainbow trout sequences rooted with the teleosts *slc16a3* sequences (Fig. S7). However, this analysis did not succed in separating clearly duplicated genes in two groups in teleosts which could help to discrimination a « a » copy and a « b » copy. We thus arbitrarily called *slc16a3a* the sequence with the accession number GSONMG00062367001 (scaffold 1168) and *slc16a3b* the sequence borne by the scaffold 2848 (GSONMG00062146001).

**Fig. S7 Pylogenetic of *slc16a3* genes.** The phylogenetic trees were built by the neighbor-joining (NJ) method. The reliability of the inferred trees was estimated by the bootstrap method with 1,000 replications. All accession numbers (from GenBank, Ensembl, or Genoscope databases) are specified in parentheses.


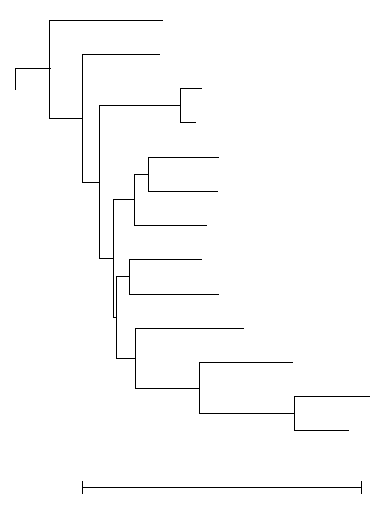


*Tetraodon nigroviridis* (ENSTNIT00000009614)

*Gasterosteus aculeatus* (ENSGACT00000002869)

*Oncorhynchus mykiss* (GSONMG00062367001) scaffold_1168-*slc16a3a*

*Oncorhynchus mykiss* (GSONMG00062146001) scaffold_2848- *slc16a3b*

*Tetraodon nigroviridis* (ENSTNIT00000017215)

*Oryzias latipes* (ENSORLT00000021569)

*Gasterosteus aculeatus* (ENSGACT00000002869)

*Danio rerio* (ENSDART00000038525)

*Danio rerio* (ENSDART00000001907)

*Lepisosteus oculatus* (ENSLOCT00000015169)

*Xenopus tropicalis* (ENSXETT00000042639)

*Homo sapiens* (ENST00000392339)

*Mus musculus* (ENSMUST00000070653)

100

49

99

43

55

31

65

25

22

54

95

100

0.2

*slc16a3*


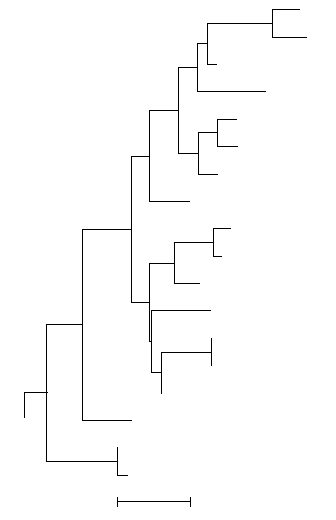


*Tetraodon nigroviridis* (ENSTNIT00000006006)

*Takifugu rubripes* (ENSTRUT00000003270)

*Gasterosteus aculeatus* (ENSGACT00000016725)

*Oryzias latipes* (ENSORLT00000023800)

*Oncorhynchus mykiss* (GSONMG00058835001) scaffold_1968-*slc2a1ab*

*Oncorhynchus mykiss* (GSONMG00008831001) scaffold_857-*slc2a1aa*

*Danio rerio* (ENSDART00000025414)

*Lepisosteus oculatus* (ENSLOCT00000004451)

*Tetraodon nigroviridis* (ENSTNIT00000021705)

*Takifugu rubripes* (ENSTRUT00000004747)

*Oryzias latipes* (ENSORLT00000009374)

*Gasterosteus aculeatus* (ENSGACT00000006436)

*Oncorhynchus mykiss* (GSONMG00006116001) scaffold_310- *slc2a1ba*

*Oncorhynchus mykiss* (GSONMG00081471001) scaffold_8-*slc2a1bb*

*slc2a1a*

*Danio rerio* (ENSDART00000137458)

*Xenopus tropicalis* (ENSXETT00000038295)

*Mus musculus* (ENSMUST00000030398)

*Homo sapiens* (ENST00000426263)

*slc2a1b*

96

31

75

69

75

71

49

93

11

23

13

47

98

61

98

0.1

***slc2a1***

**Fig. S8 Pylogenetic of *slc2a1* genes.** The phylogenetic trees were built by the neighbor-joining (NJ) method. The reliability of the inferred trees was estimated by the bootstrap method with 1,000 replications. All accession numbers (from GenBank, Ensembl, or Genoscope databases) are specified in parentheses.

Four sequences were also identified related to Slc2a1 (*Glut1*) in the rainbow trout genome whereas only two were found in other sequenced teleost genomes. Our phylogenetic analysis showed that two sequences rooted with *glut1a* teleost genes (GSONMG00008831001 and GSONMG00058835001) and the two others with the *glut1b* teleost genes (GSONMG00006116001 and GSONMG00081471001; Fig S8). The four sequences were located two by two in two distinct syntenic groups: the *tpx2-plagx* syntenic group, which included *glut1a* genes in other telelost genomes, and the *irg2-padi3-rap1gap-phc2b* group which included *glut1b* genes in other telelost genomes (Fig. S9). We thus concluded that *glut1a* and *glut1b* were retained as ohnolog pairs in the rainbow trout genome after the Ss4R and identified as *glut1aa* (scaffold_857, GSONMG00008831001), *glut1ab* (scaffold_1968, GSONMG00058835001), *glut1ba* (scaffold_310, GSONMG00006116001) and
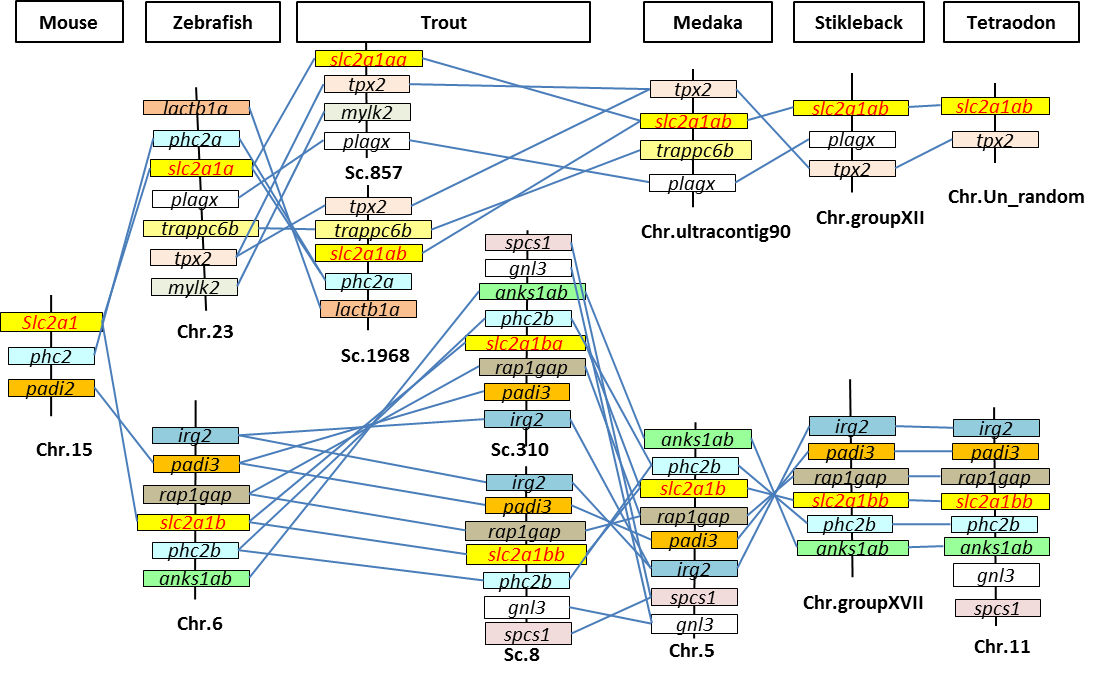
*glut1bb* (scaffold_8, GSONMG00081471001).

**Fig. S9 Syntenic analysis of *slc2a1* genes.** Conserved synteny around the *slc2a1* loci in mammals and teleosts. Data were collected with Genomicus software version 01.01, and *slc2a1* genes were annotated by ourselves according to our phylogenetic analysis following ZFIN nomenclature guidelines. Chr., chromosome; sc., scaffold.

***pfkm***

An equivalent analysis was performed for the four sequences found related to *Pfkm* in the rainbow trout genome and we also concluded that *pfkma* and *pfkmb* where retained as ohnolog pairs in the rainbow trout genome after the Ss4R and identified as *pfkmaa* (scaffold_802, GSONMG00028237001), *pfkmab* (scaffold_42194, GSONMG00035246001), *pfkmba* (scaffold_282, GSONMG00075887001) and *pfkmbb* (scaffold_185,GSONMG00069910001). Again, we thus concluded that *pfkma* and *pfkmb* where retained as ohnolog pairs in the rainbow trout genome after the Ss4R (Fig. S10, Fig. S11).


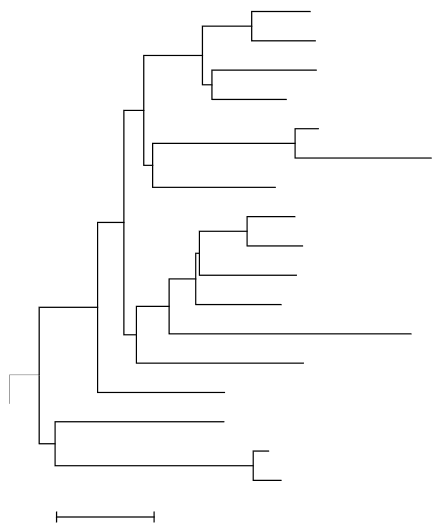


*Tetraodon nigroviridis* (ENSTNIT00000017821)

*Takifugu rubripes* (ENSTRUT00000012630)

*Gasterosteus aculeatus* (ENSGACT00000000892)

*Oryzias latipes* (ENSORLT00000002141)

*Oncorhynchus mykiss* (GSONMG00075887001) scaffold_282-*pfkmba*

*Oncorhynchus mykiss* (GSONMG00069910001) scaffold_185-*pfkmbb*

*Danio rerio* (ENSDART00000085277)

*pfkmb*

*Tetraodon nigroviridis* (ENSTNIT00000018389)

*Takifugu rubripes* (ENSTRUT00000041031)

*Oryzias latipes* (ENSORLT00000008463)

*Danio rerio* (ENSDART00000085277)

*Gasterosteus aculeatus* (ENSGACT00000011089)

*Oncorhynchus mykiss* (GSONMG00028237001) scaffold_802- *pfkmaa*

*Xenopus tropicalis* (ENSXETT00000015829)

Mus musculus (ENSMUST00000163507)

Homo sapiens (ENST00000340802)

*Lepisosteus oculatus* (ENSLOCT00000008809)

100

100

64

100

89

53

100

53

95

94

81

97

99

65

100

*pfkma*

0.05

**Fig. S10 Pylogenetic of *pfkm* genes.** The phylogenetic trees were built by the neighbor-joining (NJ) method. The reliability of the inferred trees was estimated by the bootstrap method with 1,000 replications. All accession numbers (from GenBank, Ensembl, or Genoscope databases) are specified in parentheses.


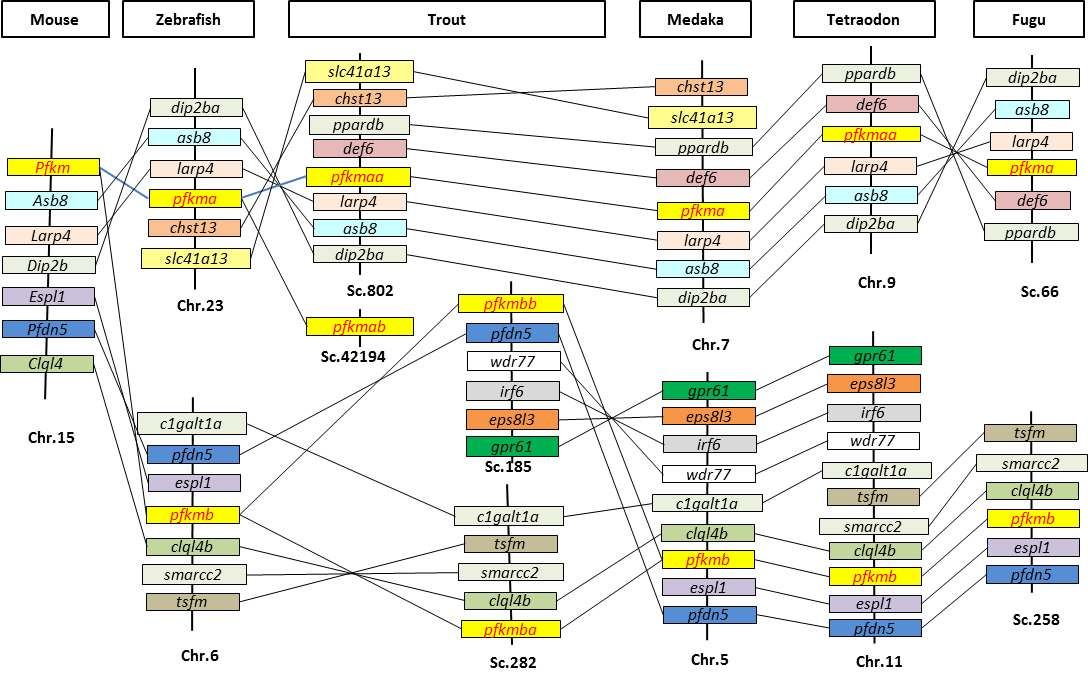
**Fig. S11 Syntenic analysis of *pfkm* genes.** Conserved synteny around the *pfkm* loci in mammals and teleosts. Data were collected with Genomicus software version 01.01, and *pfkm* genes were annotated by ourselves according to our phylogenetic analysis following ZFIN nomenclature guidelines. Chr., chromosome; sc., scaffold.

***slc2a2***

Two sequences were identified related to *Slc2a2* but bore by very short scaffolds (49682 and 9131) forbiding us to provide a relevant syntenic analysis. We thus performed a phylogenetic analysis which showed that both sequences rooted with the teleost *slc2a2* genes (Fig. S12). We annotated arbitrarily the sequences as *slc2a2a* (scaffold_49682, GSONMG00024093001) ans *slc2a2b* (scaffold_9131, GSONMG00057853001).


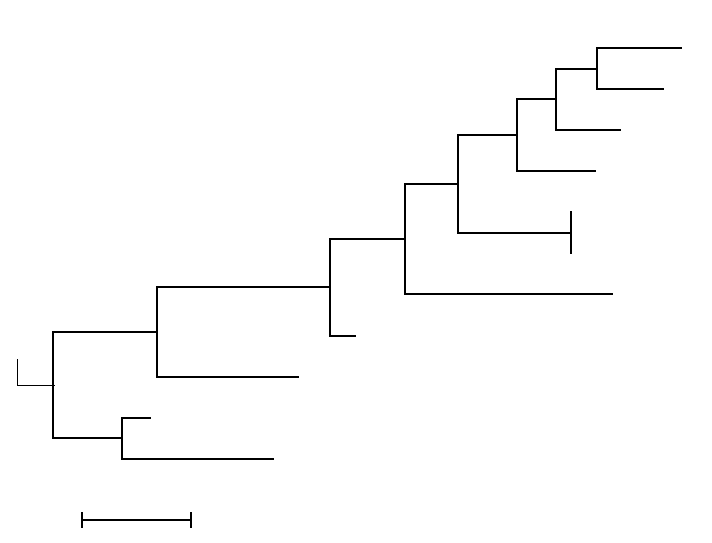


*Tetraodon nigroviridis* (ENSTNIT00000009614)

*Takifugu rubripes* (ENSTRUT00000030535)

*Gasterosteus aculeatus* (ENSGACT00000014568)

*Oryzias latipes* (ENSORLT00000014510)

*Oncorhynchus mykiss* (GSONMG00024093001) scaffold_49682-*slc2a2a*

*Oncorhynchus mykiss* (GSONMG00057853001) scaffold_9131-*slc2a2b*

*Danio rerio* (ENSDART00000078639)

*Lepisosteus oculatus* (ENSLOCT00000008895)

*Xenopus tropicalis* (ENSXETT00000020096)

*Homo sapiens* (ENST00000314251)

*Mus musculus* (ENSMUST00000029240)

69

44

63

53

99

29

82

37

87

0.1

*slc2a2*

**Fig. S12 Pylogenetic of *slc2a2* genes.** The phylogenetic trees were built by the neighbor-joining (NJ) method. The reliability of the inferred trees was estimated by the bootstrap method with 1,000 replications. All accession numbers (from GenBank, Ensembl, or Genoscope databases) are specified in parentheses.

***pkm***

Four sequences related to Pkm were found in the rainbow trout genome. We were not able to build a phylogenetic tree nor a relevant syntenic analysis as the protein sequences deduced from the Genoscope database were too short. We thus based our annotation on relationship given by the software Genomicus and by blasting the sequences against the zebrafish genome to confirm our annotation. The zebrafish *pkma* (chromosome 18) gene grouped in Genomicus with the genes GSONMG00052888001 (scaffold_1077) and GSONMG00039304001 (scaffold_67421). As these two sequences matched on the zebrafish *pkma* when blasting them against the zebrafish genome, we thus annotated them as *pkmaa* and *pkmab*, respectively. GSONMG00032476001 (scaffold_11937) and GSONMG00050270001 (scaffold_70255) grouped with the zebrafish *pkmb* and were found to have the highest score of hit with pkmb when blasted against the zebrafish genome. These two sequences were thus annotated *pkmba* and *pkmbb*, respectively.

**Supplementary Table S1.** Primer sequences and accession numbers for qPCR analysis

|  | | | |
| --- | --- | --- | --- |
| Gene | Forward Primer(5'-3') | Reverse Primer (5'-3') | Genoscope Accession Number |
| *hif1αb1 (scaffold_1551)* | CCTCACCCTTAGCTACACTGAT | AACTTTCTCTTGCGCTGTGAG | GSONMG00056387001 |
| *hif1αb2 (scaffold_3)* | CCCAACCCCTAGAGTGCTC | TGGTGAGTAAGGAAGCAGGG | GSONMG00076681001 |
| *egln3a (scaffold_148)* | GAACAAGAACTGGAACGCAG | GCATATGACGGCAGCACC | GSONMG00067638001 |
| *egln3b (scaffold_268)* | CAACAAAAACTGGAACCCCC | TCTGACCAAAAGAACAGCAGTC | GSONMG00074971001 |
| *pdk1 (scaffold_18)* | GATCCGAAACCGTCACAATG | TTTACCCTCACCTTCCCACC | GSONMG00069580001 |
| *ldhaa (scaffold_755)* | GTGTTTCTCAGCGTTCCCTG | GTTACAGAAGGGCACACAG | GSONMG00016404001 |
| *ldhab (scaffold_824)* | GTGTTCCTCAGTGTGCCATG | TTGCTGATAAATTAACCCTCCG | GSONMG00025898001 |
| *slc16a3a (scaffold_1168)* | TAGTGATGTCAAGGCACCAGAT | CACTCCGAACTCCCTGATCAAC | GSONMG00062367001 |
| *slc16a3b (scaffold_2848)* | GAGTTGCAGGCTGTAGACC | GCTCACCACAAACACAGGG | GSONMG00062146001 |
| *slc2a1aa/glut1aa (scaffold_857)* | CCAACTGGTCGGCTAACTTC | TGACTGTCCGGCCTCATGA | GSONMG00008831001 |
| *slc2a1ab/glut1ab (scaffold_1968)* | CCGCTTCATCGTGGGACTT | ACCTGTGCCATGAGGATTCC | GSONMG00058835001 |
| *slc2a1ba/glut1ba (scaffold_310)* | GGCTGGCTTCTCTAACTGGACC | TCTCCCCCAGTGCCAGCT | GSONMG00006116001 |
| *slc2a1bb/glut1bb (scaffold_8)* | GTTTGTGGTGGAGCGTGCT | AGGACATCCATGGCAGCTTG | GSONMG00081471001 |
| *slc2a2a/glut2a (scaffold_49682)* | GACAGGCACTCTAACCCTAG | CTTCCTGCGTCTCTGTACTG | GSONMG00024093001 |
| *slc2a2b/glut2b (scaffold_9131)* | CTATCAGAGAACGGTACAGGG | CAGGAAGGATGACACCACG | GSONMG00057853001 |
| *slc2a4a/glut4a (scaffold_140)* | CATCTTTGCAGTGCTCCTTG | CAGCTCTGTACTCTGCTTGC | GSONMG00067238001 |
| *slc2a4b/glut4b (scaffold_1045)* | TCGGCTTTGGCTTCCAATATG | GTTTGCTGAAGGTGTTGGAG | GSONMG00016098001 |
| *pfkmaa (scaffold_802)* | GTCAGTCTGTCCGGTAACCA | ATCTGGAGGGTTGATGTGGG | GSONMG00028237001 |
| *pfkmab (scaffold_42194)* | TCAGCGGAGGAGGCTAATC | GACTCTGTGCAGTAGTCGTG | GSONMG00035246001 |
| *pfkmba (scaffold_282)* | CTGGGCATGAAAAGGCGAT | GTCTTCTTGATGATGTGCTCCA | GSONMG00075887001 |
| *pfkmbb (scaffold_185)* | CGGTCGTATCTTTGCCAACATG | TGTCCATTTCCACAGTGTCATATT | GSONMG00069910001 |
| *pkmaa (scaffold_1077)* | ACATTGCCCCCTACAGTTAC | AAGTGGAAATGAATGGGACGT | GSONMG00052888001 |
| *pkmab (scaffold_67421)* | TGCTGAGGGCAGTGACGTA | AGCTCCTCAAACAGCTGTCTG | GSONMG00039304001 |
| *pkmba (scaffold_11937)* | CAAGCCTGCCAACGATGTC | CAAGGAACAAGCACAACACG | GSONMG00032476001 |
| *pkmbb (scaffold_70255)* | CAACTGTGACGAGAAGCACC | GAGCCCAGAGTACCACCATT | GSONMG00050270001 |

**Supplementary Table S2**. **Formulation and proximate composition of the two experimental diets used in this experiment.** HC, high carbohydrate diet; NC, no carbohydrate diet.^1^ Sopropeche, Boulogne-sur-Mer, France; ^2^ Sopropeche, Boulogne-sur-Mer,France; ^3^ gelatinized corn starch (Roquette, Lestrem, France); ^4^ D-(+)-glucose (Sigma-Aldrich, G7021); ^5^ supplying (kg^−1^ diet): 60 IU DL-α-tocopherol acetate, 5 mg sodium menadione bisulphate, 15,000 IU retinyl acetate, 3000 IU DL-cholecalciferol, 15 mg thiamine, 30 mg riboflavin, 15 mg pyridoxine, 0.05 mg vitamin B_12_, 175 mgnicotinic acid, 500 mgfolic acid, 1000 mginositol, 2.5 mg biotin, 50 mg calcium panthothenate and 2000 mg choline chloride; ^6^ supplying (kg^−1^diet): 2.15 g calcium carbonate (40% Ca), 1.24 g magnesium oxide (60% Mg), 0.2 g ferric citrate, 0.4 mgpotassium iodide (75% I), 0.4 g zinc sulphate (36% Zn), 0.3 g copper sulphate (25% Cu), 0.3 g manganese sulphate (33% Mn), 5 g dibasic calcium phosphate (20% Ca, 18% P), 2 mg cobalt sulphate, 3 mg sodium selenite (30% Se), 0.9 g potassium chloride and 0.4 g sodium chloride; and ^7^ Louis François, Marne-la-Vallée, France.

| Ingredient g/100g diet | HC | NC |
| --- | --- | --- |
| Fish meal^1^ | 22.8 | 91.2 |
| Fish oil^2^ | 13.2 | 4.8 |
| Starch^3^ | 40.0 | 0.0 |
| Glucose^4^ | 20.0 | 0.0 |
| Vitamin mix^5^ | 1.0 | 1.0 |
| Mineral mix^6^ | 1.0 | 1.0 |
| Alginate^7^ | 2.0 | 2.0 |
| *Analysed composition* |  |  |
| Dry matter (DM,% diet) | 90.1 | 93.4 |
| Crude protein (% DM) | 18.0 | 63.0 |
| Crude lipids (% DM) | 15.8 | 16.1 |
| Gross energy (kJ g^-1^ DM) | 21.6 | 22.4 |
| Ash (% DM) | 5.0 | 15.6 |
| Carbohydrates (% DM) | 48.8 | 2.4 |
|  |  |  |
